# In silico analysis of missense mutations in exons 1–5 of the *F9* gene that cause hemophilia B

**DOI:** 10.1186/s12859-019-2919-x

**Published:** 2019-06-28

**Authors:** Lennon Meléndez-Aranda, Ana Rebeca Jaloma-Cruz, Nina Pastor, Marina María de Jesús Romero-Prado

**Affiliations:** 1 Doctorado en Genética Humana, Centro Universitario de Ciencias de la Salud, Universidad de Guadalajara, C.P, 44340 Guadalajara, Jalisco México; 2 División de Genética, Centro de Investigación Biomédica de Occidente, Instituto Mexicano del Seguro Social (IMSS), Jalisco, C.P, 44340 Guadalajara, Mexico; 3 Centro de Investigación en Dinámica Celular, CIDC, Universidad Autónoma del Estado de Morelos, Cuernavaca, Mexico; 4 Departamento de Fisiología, Centro Universitario de Ciencias de la Salud, Universidad de Guadalajara, C.P, 44340 Guadalajara, Jalisco México

**Keywords:** *F9* exons 1–5, In silico analysis, Genotype-phenotype correlation, Hemophilia B

## Abstract

**Background:**

Missense mutations in the first five exons of *F9*, which encodes factor FIX, represent 40% of all mutations that cause hemophilia B. To address the ongoing debate regarding in silico identification of disease-causing mutations at these exons, we analyzed 215 missense mutations from www.factorix.org using six in silico prediction tools, which are the most common used programs for analysis prediction of impact of mutations on the protein structure and function, with further advantage of using similar approaches. We developed different algorithms to integrate multiple predictions from such tools. In order to approach a structural analysis on FIX we performed a modeling of five selected pathogenic mutations.

**Results:**

SIFT, PolyPhen-2 HumDiv, SNAP2, and MutationAssessor were the most successful in identifying true non-causative and causative mutations. A proposed function integrating these algorithms (*wgP4*) was the most sensitive (90.1%), specific (22.6%), and accurate (87%) than similar functions, and identified 187 variants as deleterious. Clinical phenotype was significantly associated with predicted causative mutations at all five exons. However, PolyPhen-2 HumDiv was more successful in linking clinical severity to specific exons, while functions that integrate 4–6 predictions were more successful in linking phenotype to genotypes at the light chain (exons 3–5). The most important value of integrating multiple predictions is the inclusion of scores derived from different approaches. Modeling of protein structure showed the effects of pathogenic nsSNPs on structure and function of FIX.

**Conclusions:**

A simple function that integrates information from different in silico programs yields the best prediction of mutated phenotypes. However, the specificity, sensitivity, and accuracy of genotype-phenotype predictions depend on specific characteristics of the protein domain and the disease of interest as we validated by the structural analysis of selected pathogenic *F9* mutations. The proposed function integrating algorithm (*wgP4*) might be useful for the analysis of nsSNPs impact on other genes.

**Electronic supplementary material:**

The online version of this article (10.1186/s12859-019-2919-x) contains supplementary material, which is available to authorized users.

## Background

Hemophilia B is a recessive X-linked disorder characterized by defective function or loss of the coagulation factor IX due to mutations in the gene *F9*, of which 40% cluster in exons 1–5 [1]. By international consensus, hemophilia B is considered severe when residual factor IX activity is < 1%, moderate when levels are between 1 and 5%, and mild when levels are > 5% [2]. The precursor contains an N-terminal prepro-leader sequence consisting of a signal peptide (exon 1) and a propeptide (exon 2), followed by a light chain that contains a gamma-carboxyglutamic (Gla) domain (exon 3), two epidermal growth factor-like domains (exons 4 and 5), a linker (exon 6), an activation peptide, and a C-terminal heavy chain containing the catalytic domain (exons 7 and 8) [3].

In early translation, the signal peptide directs the polypeptide towards the endoplasmic reticulum, and is then eliminated [4]. Subsequently, the propeptide triggers the carboxylation of the Gla domain by forming a binding site for gamma-glutamyl carboxylase [5, 6]. The ensuing removal of the signal and propeptide generates the fully functional mature protein [7]. Factor IX can be activated both by factor XIa and by the tissue factor/factor VIIa complex, which eliminate the activation peptide to generate the light chain and the heavy chain [8]. In the presence of calcium, the Gla domain undergoes conformational changes to interact with the plasma membrane of active platelets [9]. Similarly, binding of calcium to the EGF-1 domain elicits conformational changes that enable interaction with the tissue factor/factor VII complex [10], and that enable the EGF-2 and proteolytic domains to form the factor IXa/factor VIIIa complex, which, in turn, is critical to the activation of factor X at platelet membranes during coagulation [11, 12].

Thus, it is important to identify factor IX mutations that prevent protein-protein interactions and subsequent clotting. Recently, a large number of mutations of unknown functional significance were described [13], although these mutations are difficult and time-consuming to characterize in vitro [14]. On the other hand, computational analysis has become reliable as a tool to predict the possible biological effects of mutations, and may help focus resources on those that warrant exhaustive and functional analysis. To achieve the best correlation between clinical phenotype and specific mutations in *F9*, biochemical and molecular parameters have been combined with bioinformatics data [15, 16]. Similarly, we have now analyzed mutations in *F9* exons 1–5 through multiple bioinformatics tools to assess the concordance between predicted effects and reported clinical severity. We found that a mutation predicted as deleterious may be associated with a severe clinical phenotype depending on the domain in which it occurs. In addition, the data suggest that it is not necessary to use a large number of programs to accurately predict the effects of a mutation.

## Methods

The factor IX amino acid sequence was obtained from UniProt [17], and numbered according to Yoshitake et al. [18].

### Selection of missense mutations and in silico tools

F9 mutations are referred on different databases included in the Coagvdb database (info.vit.ac.in/CoagVdb/index.html), from which, missense mutations in *F9* exons 1–5 were obtained from www.factorix.org [1]. Non-synonymous single nucleotide polymorphisms (nsSNPs) in *F9* coding regions were also collected from the NCBI single nucleotide polymorphism database with access number NP_000124.1 [13]. The nsSNPs were analyzed using multiple online bioinformatics tools to obtain a reliable in silico prediction of deleterious effects, if any (Table 1). We chose SIFT, PolyPhen2, PROVEAN, MutationAssessor and Panther as they are commonly used tools available for free, using a similar approach (sequence conservation), applying various methods to calculate sequence conservation. In addition, we chose SNAP2 which, like PolyPhen2, integrates characteristics based on sequence and structure using an automatic learning approach (machine learning) to categorize variants as benign or damaging (Table 1).

**Table 1:** Bioinformatics tools for in silico analysis

Program	Based on	Prediction	Score	Functional impact (reference)	Available at
Poly Phen 2*	Sequence- and structure-based approach	Benign	< 0.5	On the structure and function of a human protein [19]	http://genetics.Bwh.harvard.edu/pph2/index.shtml
		Possibly damaging	≥0.5		
		Probably damaging			
SIFT	Sequence-based approach	Tolerated	≥0.05	On protein function and the physiochemical properties of AA [20].	http://sift.jcvi.org/
		Damaging	< 0.05		
PANTHER	Sequence-based approach	Probably benign	0 to −3	Estimates the likelihood of a particular nonsynonymous coding SNP causing a functional impact on the protein [21].	http://www.pantherdb.org/tools/csnpScoreForm.jsp
		Possibly damaging	<−3		
		Probably damaging			
MutationAssessor	Sequence-based approach	neutral	≤0.8	On the substitution of AA in the protein by assessing evolutionary conservation [22].	http://mutationassessor.org
		low impact	0.8 to < 1.9		
		medium impact	1.9 to ≤3.5		
		high impact	> 3.5		
PROVEAN	Sequence-based approach	Neutral	> − 2.5	On the biological function of a protein [23].	http://provean.jcvi.org/index.php
		Deleterious	<−2.5		
SNAP2	Sequence- and structure-based approach	Neutral	100	On the secondary structure and compares the solvent accessibility of the wild and mutated protein [24].	https://rostlab.org/services/snap2web
		Effect	− 100		

*, “HumDiv” is the default Classifier model used by probabilistic predictor; it is preferred for evaluating rare alleles, dense mapping of regions identified by genome-wide association studies, and analysis of natural selection. “HumVar” is better suited for diagnostics of Mendelian diseases, which requires distinguishing mutations with drastic effects from all the remaining human variation, including abundant mildly deleterious alleles

**Fig. 1:**
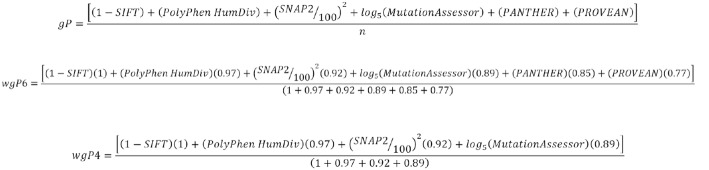
Formulas for combined predictions. (1 – SIFT), as SIFT scores are inverse to PolyPhen-2 scores, they were scaled by subtracting from 1. PolyPhen, score obtained from PolyPhen-2 HumDiv. (SNAP2/100)2, SNAP2 scores may be positive and negative percentages, they were scaled to PolyPhen-2 scores by dividing by 100 and squaring. MutationAssessor, scores range from 4 to − 2. Mutations scoring below 1.9 are considered benign, and so are coded as 1. Predicted values were log-transformed at base 5 to obtain values between 0 and 1. PANTHER and PROVEAN, predictions are categorized as deleterious or benign, and are coded 1 and 0 respectively. n, number of programs used in combined analysis. In the functions *wgP6* and *wgP4*, n is substituted by the weight for each program. In B and C, predicted values in the numerator are multiplied by the weight

To improve the quality of predictions, we combined four (*wgP4*) or six (*wgP6*) programs using corresponding functions that were designed to generate binary predictions similar to PolyPhen-2, so that scores 0–0.5 were considered benign and scores between 0.5 and 1 were regarded as deleterious (Fig. 1). The functions were also designed to weight each program, so that the program with the highest accuracy was weighted 1 and all other programs were weighted proportionally (see Table 2 in Results).

**Table 2:** In silico analysis of 215 single nucleotide polymorphisms at *F9* exons 1–5

Program	Variants predicted as deleterious (%)	Variants predicted as benign (%)	Accuracy	Weight^e^
SIFT	194 (90.2)	21 (9.8)	90.2	1
PolyPhen-2 HumDiv	189 (87.9)	26 (12.1)	87.9	0.974
PolyPhen-2 HumVar	179 (83.3)	36 (16.7)		
SNAP2	184 (58.6)	31 (14.4)	85.6	0.924
MutationAssessor^a^	188 (87.4)	27 (12.6)	87.4	0.896
PANTHER	184 (85.6)	31 (14.4)	85.6	0.850
PROVEAN	176 (81.9)	39 (18.1)	81.9	0.772
*gP* ^b^	184 (85.6)	31 (14.4)		
*wgP*6^c^	184 (85.6)	31 (14.4)		
*wgP*4^d^	187 (87.0)	28 (13.0)		

^a^MutationAssessor scores mutational impact as neutral, low, medium, and high. Neutral and low impact were considered benign, while medium and high impact were considered deleterious

^b^Combined prediction

^c^Weighted combined prediction from six programs

^d^Weighted combined prediction from four programs

^e^The program with highest accuracy was weighted 1, and all other programs were weighted proportionally

### Sensitivity, specificity, and accuracy

Based on the FIX activity and secondarily, on the associated clinical phenotype reported in the consulted sources, the severity of the phenotype was categorized as severe (FIX activity 0–5%) or non-severe (FIX activity higher than 5%) [25, 26]. Predictions were classified as true positive (TP, severe phenotype predicted from a damaging mutation), false positive (FP, non-severe phenotype predicted as damaging mutation), true negative (TN, non-severe phenotype predicted as benign mutation), and false negative (FN, severe phenotype predicted as benign mutation). Sensitivity was calculated as TP/(TP + FN) × 100, specificity was calculated as TN/(TN + FP) × 100, and accuracy was calculated as (TN + TP)/(TN + FP + FN + TP) × 100.

### Statistical analysis of in silico prediction vs. phenotype

Two-tailed Pearson’s χ^2^ test or Fisher’s exact test in SPSS 20.0 [27] were used to assess the relationship between in silico prediction for each variant vs. clinical severity. *P* < 0.05 was considered statistically significant.

### Secondary structure

The FFPRED tool in PSIPRED [28] was used to analyze changes in secondary structure (alpha helix, extended strand, and random coil) and other protein properties (aliphatic index, hydrophobicity, surface area, and addition or deletion of phosphorylation sites). Secondary structure was predicted for the sequence corresponding to the signal peptide, propeptide, and the Gla, EGF-1, and EGF-2 domains.

### Tertiary structure modeling of selected mutations on the EGF domains

Using the structure of the light chain from the full FIX protein from pig (PDB ID 1PFX, chain L [29] as a template in I-TASSER (Iterative Threading Assembly Refinement) [30] we modeled the human *F9* EGF domains and C-terminal linker (residues 93 to 192) with the mutations p.Gln96Pro, p.Gly105Asp, p.Glu124Lys, p.Gln143Arg, and p.Val153Met. As this structure lacks calcium, we also modeled EGF-1 (residues 93 to 129) with the p.Gln96Pro mutation using the structure of EGF-1 from human *F9* with calcium (PDB ID [31]) as reference. All modeling attempts resulted in a single structure, with C-scores > 1.4 and TM-scores > 0.9, so, according to I-TRASSER criteria, these are well-known and very reliable models [32]. The structure of the complex between the EGF domains and the catalytic domain was obtained by superposition of the modeled EGF-2 domains with that of the human EGF-2 domain in the most recent high resolution structure of a fragment of human F9 (PDB ID 6MV4 [33]. All models were inspected in VMD [34].

## Results

### Selection of single nucleotide polymorphisms and missense mutations

We analyzed 215 missense mutations deposited at www.factorix.org for exons 1–5 in *F9*. Residual factor IX activity was obtained from associated publications. Two nonsevere mutations were noted at the signal peptide, along with 11 severe mutations. In the propeptide, 16 severe mutations were noted, along with 55, 41, and 39 severe mutations in Gla, EGF-1, and EGF-2. In addition, 16, 27, and 8 nonsevere mutations were noted in Gla, EGF-1, and EGF-2. According to severity criteria, we selected five mutations to be analyzed for changes in their tertiary structure of FIX protein.

### In silico analysis

The number of the variants predicted as deleterious by individual programs is listed in Table 2. SIFT, PolyPhen-2 HumDiv, and MutationAssessor identified the highest number of variants as deleterious, while PROVEAN, PolyPhen-2 HumVar, PARTNER, and SNAP2 identified the highest number of variants as benign. A function that weights predictions from 6 programs (*wgP6*) identified 184 variants as deleterious based on a threshold of ≥0.5. Excluding PROVEAN and PANTHER, which were less accurate, a function integrating the remaining four programs (*wgP4*) identified 187 variants as deleterious. Additional data are provided in Additional file 1: Table S1), including results from integrating all seven programs.

### Sensitivity, specificity, and accuracy

**Fig. 2:**
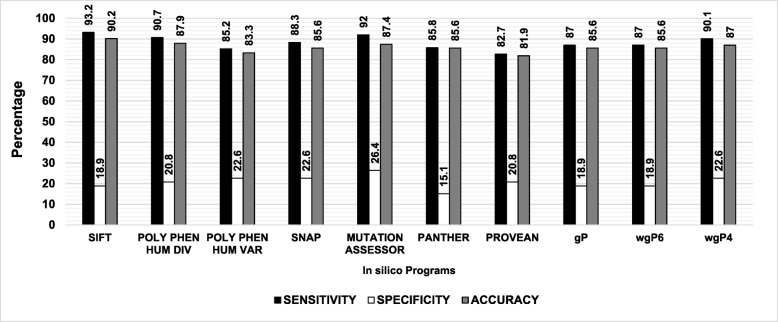
Sensitivity, specificity, and accuracy for five factor IX domains. The first five domains encoded by exons 1–5 were analyzed as one unit using individual tools. See text for more details

Analysis of mutations at all domains indicated that SIFT was the most accurate, followed by PolyPhen-2 HumDiv, SNA2P, and MutationAssessor. After integrating the scores of these four programs into *wgP4*, the accuracy was 87% (Table 2). SIFT was also the most sensitive (93.2%), but not the most specific (18.9%), while MutationAssessor was the next most sensitive (92%) and the most specific (26.4%). *wgP4* was the most sensitive (90.1%) and specific (22.6%) of combined functions (see Fig. 2).

**Fig. 3:**
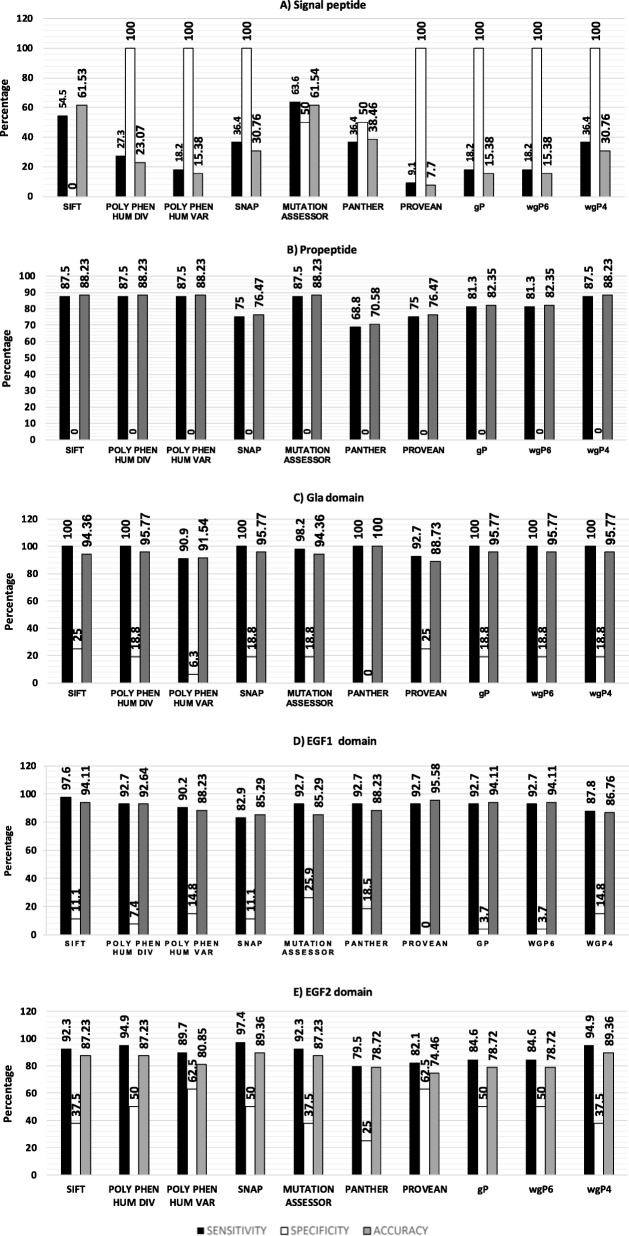
Sensitivity, specificity, and accuracy for each factor IX domain. The (**a**) signal peptide at exon 1, (**b**) propeptide at exon 2, (**c**) Gla domain at exon 3, (**d**) EGF-1 domain at exon 4, and (**e**) EGF-2 domain at exon 5 were analyzed by individual tools. See text for more details

As shown in Fig. 3, only few mutations have been reported in the first two domains (exons 1–2), most of which are known to cause severe hemophilia B. Specificity was 100% for PolyPhen-2 HumDiv, PolyPhen-2 HumVar, SNAP2, PROVEAN, and the three combined functions. However, SIFT classified the only two cases of nonsevere phenotype as deleterious (0% specificity). MutationAssessor was the most sensitive (63.6%) and accurate (61.54%), while *wgP4* was the most specific (36.4%) and accurate (30.8%) of combined functions. Because mutations analyzed in exon 2 (propeptide domain) were all severe, specificity was 0% in all cases, although sensitivity was highest (87.5%) in SIFT, PolyPhen-2 HumDiv and HumVar, MutationAssessor, and the combined function *wgP4*. The proportions of mutations causing severe phenotype were of 77.5 and 83% for Gla and EGF-2 domains although such mutations were less common in EGF-1 (60.3%). Of note, only PANTHER and PROVEAN failed to identify true negatives in exon 3 and 4, respectively.

### Association between in silico analysis and phenotype

As an grouped analysis, based on analysis by SIFT, PolyPhen-2 HumDiv, MutationAssessor, and *wgP4*, deleterious mutations at all five domains, as well as in Gla, EGF-2, and the light chain (exons 3–5) were significantly associated to with severe phenotype (*P* < 0.05) (residual factor IX activity 0–5%). A significant association (P < 0.05) was also observed between severe phenotype and mutations in the light chain that were predicted to be deleterious by SNAP2. However, the correlation between phenotype and light chain genotype was strongest by integrating 4–6 programs (Table 3). Finally, mutations in Gla that were predicted to be deleterious by all programs except PolyPhen-2 HumVar and PROVEAN were also significantly correlated with clinical phenotype.

**Table 3:** Association between clinical severity and in silico analysis of single nucleotide polymorphisms^c^

	Leader sequence^d^ + Light chain^e^			Gla Domain			EGF-2 Domain			Light chain^e^		
	χ^2^	*P* (two-tailed)^a^	*P* (two-tailed)^b^	χ^2^	*P* (two-tailed)^a^	P (two-tailed)^b^	χ^2^	*P* (two-tailed)^a^	*P* (two-tailed)^b^	χ^2^	*P* (two-tailed)^a^	*P* (two-tailed)^b^
SIFT	6.610^a^	**0.010**	0.016	14.571^a^	0.000	**0.002**	5.296^a^	0.021	0.053	14.734^a^	**0.000**	**0.000**
PolyPhen-2 HumDiv	4.964^a^	**0.026**	0.049	10.767^a^	0.001	**0.010**	12.002^a^	0.001	**0.005**	10.339^a^	**0.001**	**0.003**
PolyPhen-2 HumVar	1.755^a^	0.185	0.206	0.129^a^	0.719	1.000	11.703^a^	0.001	**0.004**	3.401^a^	0.065	0.081
SNAP2	3.854^a^	0.050	0.070	10.767^a^	0.001	**0.010**	15.713^a^	0.000	**0.002**	7.927^a^	**0.005**	**0.010**
Mutation Assessor	12.300^a^	**0.000**	0.001	6.684^a^	0.010	**0.034**	5.296^a^	0.021	0.053	15.903	**0.000**	**0.000**
PANTHER	0.026^a^	0.872	0.826				0.080^a^	0.778	1.000	1.317^a^	0.251	0.272
PROVEAN	0.324^a^	0.569	0.545	3.896^a^	0.048	0.070	6.930^a^	0.008	*0.018*	1.809^a^	0.179	0.213
*gP*	1.128^a^	0.288	0.367	10.767^a^	0.001	**0.010**	4.749^a^	0.029	0.051	3.626^a^	0.057	0.084
*wgP6*	1.128^a^	0.288	0.367	10.767^a^	0.001	**0.010**	4.749^a^	0.029	0.051	3.626^a^	0.057	0.084
*wgP4*	5.745^a^	**0.017**	0.032	10.767^a^	0.001	**0.010**	7.318^a^	0.007	**0.029**	9.272^a^	**0.002**	**0.008**

^a^By Pearson’s χ^2^ test

^b^By Fisher’s exact test

^c^Severe vs. non-severe phenotypes

^d^Signal peptide + propeptide

^e^Gla + EGF-1 + EGF-2

**Fig. 4:**
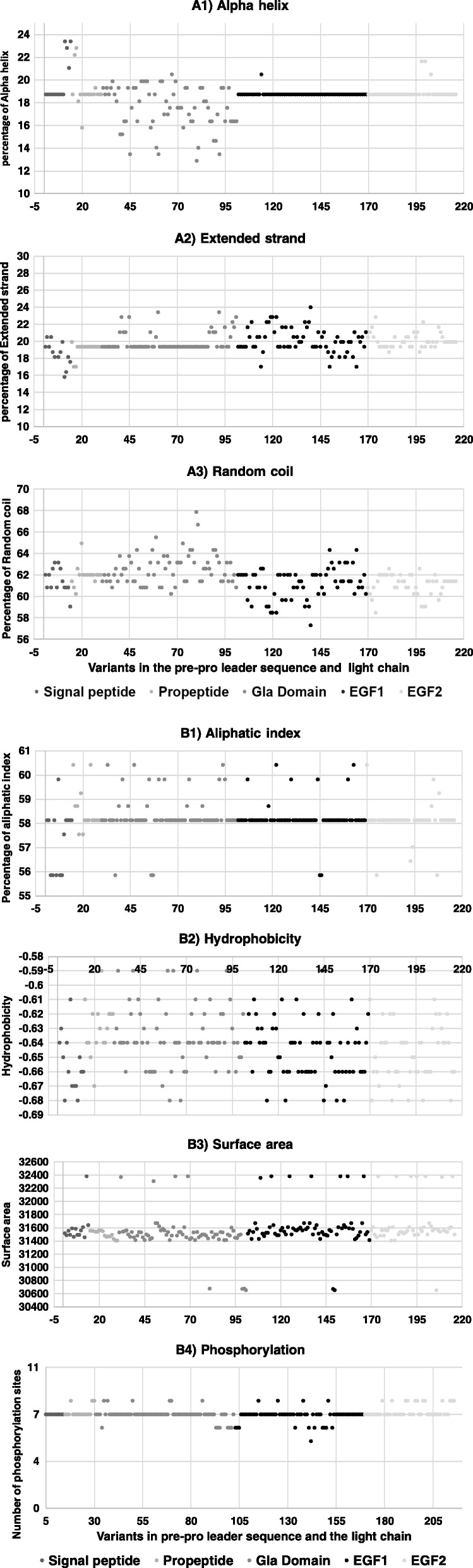
Analysis of factor IX secondary structure by the FFPRED tool in PSIPRED. Analysis of predicted changes in (**a**) percentage alpha helix, extended strand, and random coil, as well as in (**b**) aliphatic index, hydrophobicity, surface area, and addition or deletion of phosphorylation sites. Domains are depicted in different shades of gray

In order to test the possible corroboration of changes in secondary structure due to the 215 amino acid changes, we recapitulated the effects of mutations on hydrophobicity, surface area, aliphatic index, percentage of alpha helix, extended strand, random coil, and number of phosphorylation sites (Fig. 4). The prediction for the sequence corresponding to the signal peptide, propeptide, and the Gla, EGF-1, and EGF-2 domains, was made by using PSIPRED analysis.

### Association between predicted structural impact and phenotype

**Fig. 5:**
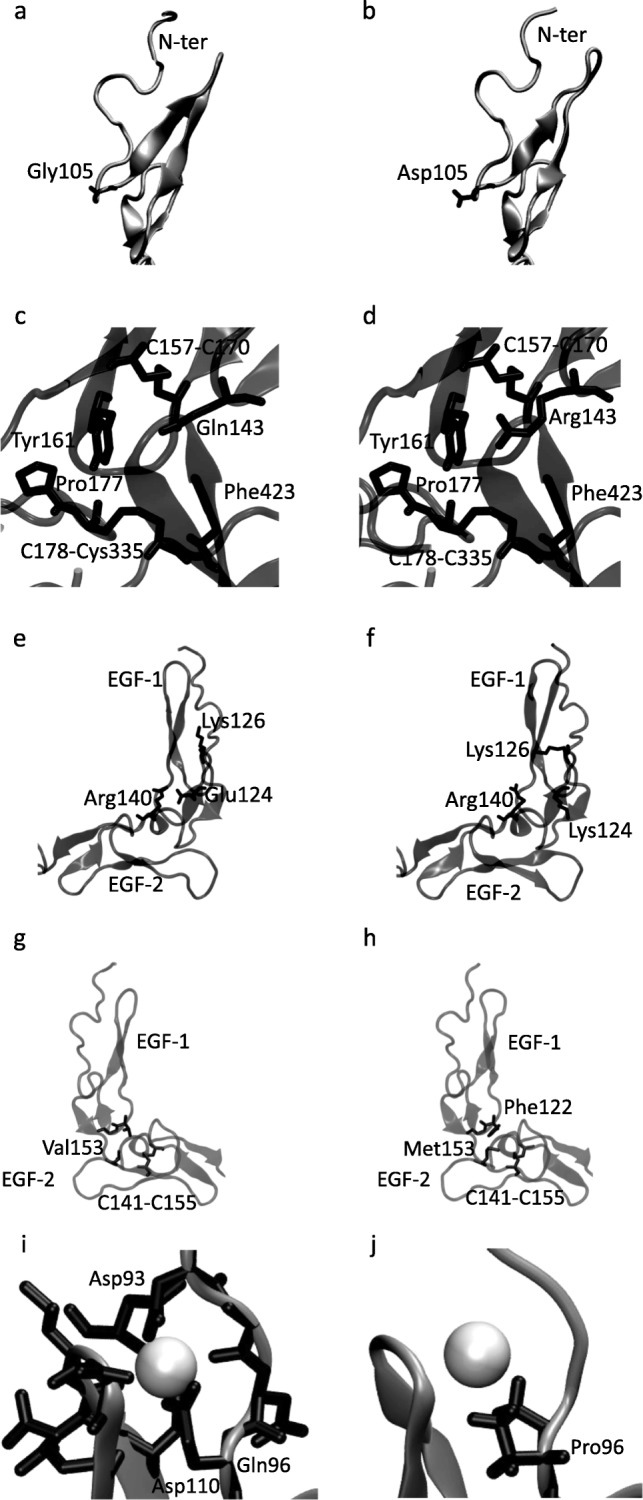
Comparison of the local environment of severe and mild mutations in the EGF domains of FIX. The protein backbone is shown in silver ribbons, interacting amino acids as a black licorice and the calcium ion as a white sphere. **a**, **c**, **e**, **g**, **i** correspond to wild type FIX. **b**, **d**, **f**, **h**, **j** correspond to mutant FIX. **a** Location of Gly105 in EGF-1 (from PDB ID 1PFX); the N-terminus of the domain is labeled. **b** Location of Asp105 in EGF-1; the N-terminus of the domain is labeled **c** Neighboring residues for Gln143 (from PDB ID 6MV4), labeled. **d** Neighboring residues for Arg143, clashing with the disulfide bond between Cys157 and Cys170, Tyr161 and Phe423. **e** Salt bridge between Glu124 in EGF-1 and Arg140 in EGF-2; neighboring positive residue also labeled (from PDB ID 1PFX). **f** Group of nearby positive charges in the Glu124Lys mutant. **g** Selected residues close to Val153 (from PDB ID 1PFX). **h** Residues that clash with Met153. **i** Residues coordinating the calcium ion in EGF-1; the residues that contribute their side chains are labeled (from PDB ID 1EDM). **j** Location of Pro96 as a first coordination shell residue for calcium

In order to explore the consequences of selected mutations on FIX structure and protein-protein interactions, we modeled four severe mutations (p.Gln96Pro, p.Glu124Lys, p.Gln143Arg, and p.Val153Met) and a mild one (p.Gly105Asp). A comparison of the local structure around the mutation site, in the wild-type and mutant versions, is shown in Fig. 5.

The Gly105Asp mutation happens in an exposed loop and does not have any negative charges nearby that would repel it (Fig. 5a and b), explaining why it is apparently well tolerated.

One of the severe mutations lies at the interface between the light chain and the catalytic domain. Gln143 fits snugly against Tyr161 and the disulfide bond formed by C157 and Cys170 (Fig. 5c). As Arginine is larger than Glutamine, Arg143 clashes against Tyr161 and the disulfide from the same domain, and with Phe208 (Phe423 considering the full protein) from the catalytic domain (Fig. 5d). Relieving this clash by displacing Tyr161 results in a new clash with Pro177, which could affect the position of the C-terminal linker and the interdomain disulfide bond with the catalytic domain (Cys178 from the linker and Cys122 (Cys335 considering the full protein) from the catalytic domain).

Two of the severe mutations lie at the interface between EGF-1 and EGF-2. Glu124 (Fig. 5e) forms a conserved salt bridge with Arg140 in EGF-2, stabilizing the interaction between the domains. Mutation of Glutamate to Lysine results in a predominantly positive interface between the two domains (Fig. 5f), which is likely to alter the angle of interaction. Located in the loop below this salt bridge, Val153 (Fig. 5g) fits in a densely packed cavity at the interface between EGF-1 and EGF-2; Met153 (Fig. 5h) cannot fit properly in the same space, bumping against one of the disulfide bonds of EGF-2 (Cys155 and Cys141) and against a loop in EGF-1 (Phe122 and Gly123), potentially altering the angle between both domains.

The remaining severe mutation lies at the calcium-binding site of EGF-1. The side chain of Gln96 is part of the coordination shell of the calcium ion (Fig. 5i), so the mutation to Proline (Fig. 5j) eliminates one of the ligands and is likely to decrease affinity for the ion.

## Discussion

In this study, we analyzed specific, interacting protein domains that impact the activity of factor IX. Accordingly, six freely available bioinformatics tools were used to find potentially deleterious missense mutations and single nucleotide polymorphisms. Sensitivity, specificity, and accuracy were assessed based on observed clinical phenotypes. Also, we considered the secondary and tertiary structures analysis in an attempt to enhance the approaches to a possible correlation between in silico prediction and clinical phenotypes. These approaches were integrated in the molecular modeling of F9 selected mutations in an attempt to corroborate the correlation between in silico prediction and clinical phenotype.

### Reliability of in silico predictions

The deleterious effects of missense mutations in *F9* gene, especially at the first five domains of the precursor protein product, have been studied in silico in several studies. In this study, we integrated results from several bioinformatics tools to enhance the quality of predictions. The six tools integrated were selected not only based on performance, but also for the complementarity or diversity of approach to the analysis of an amino acid sequence. Previously, Ou et al. [35] reports a total of 285 mutations with a 52% of concordance between predicted deleterious mutations in *IDUA* gene made by SITF and Poly Phen. In contrast, the concordance dropped to 9.83% when seven programs were used. Similarly, we found that concordance was 85.6% (*n* = 184 mutations) using SIFT and PolyPhen-2, but 67.4% using all six programs and or various combinations thereof (data not shown). These results imply that prediction quality does not necessarily improve by using a larger number of bioinformatics tools, but by proper selection of programs that analyze properties closely related to the biological function of the gene and to the associated trait.

We have formulated a straightforward way to integrate programs (gwP4) by which to generate reliable predictions. Similar tools have been described, including Condel [36], Meta-SNP [37], PON-P2 [38], and PredictSNP [39]. Condel combines SIFT, PolyPhen-2, MutationAssessor, and MAPP. Notably, concordance was high (89%) between Condel and PROVEAN, especially when mutations are predicted to be deleterious [40]. Similarly, we found that predictions from gwP4 were 84.2% concordant to results from SIFT and PolyPhen-2, and 81.4% concordant to predictions from PROVEAN (data not shown). These results highlight the notion that fewer programs may be better to identify a mutation as deleterious.

The most important innovation from this work about the integration of predictions into wgP4 is the inclusion of a wide variety of scores and predictions from different programs, yielding dichotomized results. However, this analysis might mask intermediate phenotypes and is therefore suitable only for categorical phenotypes. On the other hand, this approach focuses on coding regions and nonsynonymous mutations, which represent more than 60% of all missense mutations described for F9, but excludes mutations in introns and promoters, as well as synonymous mutations and mutations that alter RNA stability, all of which have also been associated with coagulation diseases. Therefore, it may be necessary to consider parameters such as RNA stability to predict the effect of synonymous mutations on protein synthesis [15, 16].

### Sensitivity, specificity, and accuracy

Since in silico programs have variable sensitivity, specificity, and accuracy, one or two programs may not be sufficient to predict the phenotypic effect of a mutation or single nucleotide polymorphism. Indeed, we observed that sensitivity, specificity, and accuracy depend on the protein domain. For example, very few mutations (*n* = 29/215) have been reported in the first two N-terminal domains in factor IX (signal peptide and propeptide), most of which (93.1%) have been linked to severe phenotypes [1]. The signal peptide is eminently functional, but its genetic variability provides some “flexibility” to accommodate certain genetic variants, e.g., nonconservative amino acid changes, without affecting function. Strikingly, most programs identified all true negatives (high specificity, compare Fig. 2 with Fig. 3), but only few true positives (low sensitivity, compare Fig. 2 with Fig. 3). Accordingly, accuracy was remarkably low. This result implies that if homologous sequences for a specific gene are insufficiently informative or highly variable, and if function is other than eminently structural or enzymatic, in silico programs may be of limited utility [41]. On the other hand, mutations in the propeptide are more homogeneously predicted as deleterious due to lower specificity and higher sensitivity. Hence, programs with high sensitivity are probably more useful to identify true positives in this domain. Due to the proportion of severe and nonsevere phenotypes associated with mutations in the light chain (Gla + EGF-1 + EGF-2), specificity at this domain was also low, but with high sensitivity. However, accuracy was higher than 80%, so programs with high sensitivity or specificity, i.e., SIFT, PolyPhen-2 HumDiv, SNAP2, and MutationAssessor may detect true positives and negatives, respectively. Indeed, prediction quality was highest using *wgP4*, which integrates these four programs. Our results are in line with Leong et al. [42], who found that specificity, sensitivity, and accuracy in predicting mutational effects depend on the gene and the combination of analytical tools, not necessarily on the use of a large number of tools.

### Association between prediction and clinical phenotype

As an grouped analysis, 215 mutations in the first five exons showed significant association to the clinical severity of hemophilia B based on analysis by SIFT, PolyPhen-2 HumDiv, MutationAssessor, SNAP2 (*P* ≤ 0.05), and *wgP4* (*P* = 0.017), but this association was not significant for mutations in EGF-1, as well as in the signal peptide and propeptide. Hoffman [43] describes cellular coagulation as a series of phases that depend on interactions between enzymes, cofactors, proteins, and phospholipids. During the amplification phase, factor IX is activated by the tissue factor/factor VIIa complex or by factor XIa. In turn, factor IXa and its cofactor factor VIIIa activate factor X in the propagation phase, generating large amounts of thrombin. However, hemophilia B is considered monogenic disease, and is diagnosed only based on residual factor IX activity. Hence, even in silico predictions are insufficient to determine total coagulation capacity. Accordingly, we used PolyPhen-2 to investigate hemophilia B both as a monogenic disease with rare alleles that may drastically alter protein function (HumVar), and as a complex disorder (HumDiv) modified by several genes [44, 45]. PolyPhen-2 HumDiv was found to be a better predictor of clinical severity based on mutations in a specific protein domain, a result similar to that of Martelloto et al. [46] in studies of oncogenes.

Concordance between predicted deleterious mutations and clinical phenotype was strongly variable among domains. We ascribe this to sequence variability in the signal peptide, which contains a positively charged N-terminal domain with a Lys or an Arg (domain n), a central hydrophobic domain rich in Leu (domain h), and a C-terminal hydrophilic domain (domain c) with a cleavage site [4]. The lack of context in the signal peptide appears to generate somewhat contradictory predictions, e.g., all six programs individually predicted that Leu -24Pro as deleterious, but Leu -23Pro as benign. Leu -24Pro was also predicted as deleterious by *wgP4*, in agreement with the reported phenotype. However, the Leu > Pro substitution in both cases may disrupt function, since Leu strongly tends to form alpha helices whereas Pro is often destabilizing [47]. Analysis of secondary structure also showed that these mutations affect the percentage of alpha helices, corroborating the predicted deleterious effects. On the other hand, the propeptide forms a binding site (amino acids − 18, − 17, − 16, − 15, and − 10) that interacts directly with gamma-glutamyl carboxylase [5, 48]. In particular, Phe − 16 and Ala − 10 are essential for the carboxylation of Glu residues in the Gla domain [49, 50]. Hence, mutations in the propeptide diminish or abolish the affinity for the enzyme, ultimately preventing carboxylation [51]. Nevertheless, mutations at amino acids − 18 and − 17 are associated with severe hemophilia B, but are annotated differently by several tools [52]. Hence, specialized tools such as Phobius [53] and SignalP 4.0 [54] might prove more useful in the analysis of this domain.

The EGF domains encoded by exons 4 and 5 mediate cell adhesion and ligand-receptor interactions that are important in coagulation [55]. Although these domains share similar secondary structures, only EGF-2 mutations were reliably associated with clinical phenotype when annotated by PolyPhen-2 HumDiv, PolyPhen-2 HumVar, SNAP2, and *wgP4*. On the other hand, the EGF-1 domain contained the most number of mutations predicted by all programs as deleterious, although the associated clinical phenotypes are nonsevere. The discordance in these results may be due to differences in function. In EGF-1, Asp93, Asp95, Asp110, and Tyr115 [56] are considered important for calcium binding and are associated with clinical severity. However, we found that the mutations p.Asp93Gly, p.Asp93Glu, p.Asp95Tyr, p.Asp110Gly, p.Asp110Glu, and p.Asp110Val are predicted as deleterious by all progrDisams, although the reported clinical phenotype is mild. In EGF-2, residues 88–109 form two loops directly involved in the formation of the complex that activates factor X [57]. In this case, the predicted mutational effects correlate with the clinical phenotype and are true positives.

In this sense, the five selected mutations that were modeled fall in the EGF-1 and EGF-2 domains, and some could affect the interaction of the EGF-2 domain with the catalytic domain. For these reasons, we modeled both the EGF domains on their own and in the context of the catalytic domain, using as templates the appropriate wild-type structures from either human or pig FIX.

All the SNPs are laying in EGF-1 (Gln96Pro, Gly105Asp, Glu124Lys) and EGF-2 (Gln143Arg, Val153Met) domains, from which only Gly105Asp, that lies in EGF-1 near the interface with EGF-2, does not engage in any interactions with it, so it would explain that this mutation is associated to a Hemophilia B mild phenotype.

The severe mutation Gln143Arg has repercussions at the interface between the light chain and the catalytic domain, affecting its fit with Tyr161 and the disulfide bond (Cys157 and Cys170), as well as with Phe208; this change would affect the Pro177 and the disulfide bond (Cys178 and Cys335). All these interactions are located at the surface opposite from the catalytic site of the protease domain, but may be important for correct activation by heparin and related signals [58], as they lie adjacent to a helix important for interactions with heparin.

The two mutations, Glu124Lys and Val153Met might alter the interactions proposed by Brandstetter et al. [29] with both FVIII and FX in the coagulation cascade, explaining the severe phenotype. Finally, the Gln96Pro mutation affects a calcium-binding site, decreasing affinity for the ion calcium, which is important for activation of FIX [29] and in protein-protein interactions [31], given that one of the ligands for calcium must be donated by either another protein or by a water molecule.

## Conclusions

Integration of results from selected programs into a function (*wgP4*) that generates binary predictions provides an easy approach to associate nonsynonymous mutations with severe hemophilia B that can be useful for the analysis of nsSNPs impact on other genes. Indeed, it is not necessary to use a large number of programs to predict mutational effects. Nevertheless, the specificity, sensitivity, and accuracy of genotype-phenotype predictions depend on specific characteristics of the protein domain and the disease of interest as we corroborated with a secondary and tertiary structural analysis of the effect of selected pathogenic mutations on the FIX.

## Additional file

Additional file 1: **Table S1.** Complete bioinformatics analysis (*n* = 215) and results of functions that integrate various tools. *Clinical phenotypes are severe (residual factor IX activity 0–1%), moderate (residual activity 1–5%), and mild (residual activity > 5%). **Phenotypes defined in this analysis (see text) are severe (residual activity 0–5%) and nonsevere (residual activity > 5%). *gP*, combined prediction; *wgP6*, weighted combined prediction from six programs; *wgP4*, weighted combined prediction from four programs. (DOCX 118 kb)

## Data Availability

The complete data from which we made the analysis are added as supplementary material (Data sheet, Additional file 1: Table S1). The additional information about the detailed secondary structure analysis by PSIPRED is available on request.

## Abbreviations

EGF-1: Epidermal growth factor 1
EGF-2: Epidermal growth factor 2
*F9*: Factor 9 gene
FIX: Factor IX protein
FVIII: Factor VIII protein
FX: Factor X protein
Gla: Carboxilation domain
IDUA: Iduronidase, alpha-L gene
