# Supplementary material for: In silico analysis of missense mutations in exons 1–5 of the F9 gene that cause hemophilia B
Source: BMC Bioinformatics. 2019 Jun 28;20:363. doi: 10.1186/s12859-019-2919-x (PMC6599346; doi:10.1186/s12859-019-2919-x)
Supplement: Supplementary file 1 — Table S1. Complete bioinformatics analysis (n = 215) and results of functions that integrate various tools. *Clinical phenotypes are severe (residual factor IX activity 0–1%), moderate (residual activity 1–5%), and mild (residual activity > 5%). **Phenotypes defined in this analysis (see text) are severe (residual activity 0–5%) and nonsevere (residual activity > 5%). gP, combined prediction; wgP6, weighted combined prediction from six programs; wgP4, weighted combined prediction from four programs. (DOCX 118 kb) [file 12859_2019_2919_MOESM1_ESM.docx]

Table S1. Bioinformatics *in silico* analysis and results of proposed formulas (n=215).

| FIX:C(%) | 3 Cat* | 2 Cat** |  | AA Change | SIFT | | POLY PHEN HUM DIV | | POLY PHEN HUM VAR | | SNAP | | MUTATION ASSESSOR | | PANTHER | | PROVEAN | | gP | | wgP6 | | wgP4 | | factorIX.og reference |
| --- | --- | --- | --- | --- | --- | --- | --- | --- | --- | --- | --- | --- | --- | --- | --- | --- | --- | --- | --- | --- | --- | --- | --- | --- | --- |
| <1 | Severe | S | c.8G>A | p.Arg3His | 0.54 | Tolerated | benign | 0.001 | benign | 0.001 | neutral | -95 | -0.205 | Neutral | 176 | probably begign | 0.19 | Neutral | 0.22725 | benign | 0.239133 | bening | 0.341363 | bening | Ludwig et al (1992) |
| 30 | Mild | NS | c.19A>T | p.Ile7Phe | 0.006 | Damaging | benign | 0.003 | benign | 0.002 | neutral | -10 | 1.39 | low | 176 | probably begign | -0.35 | Neutral | 0.201935 | benign | 0.219552 | bening | 0.313411 | bening | Montejo et al (1999) |
| <1 | Severe | S | c.50T>A | p.Ile17Asn | 0.002 | Damaging | possibly damaging | 0.626 | benign | 0.243 | effect | 52 | 1.935 | medium | 324 | possibly damaging | -2.26 | Neutral | 0.550758 | damaging | 0.567749 | damaging | 0.586404 | damaging | Chen et al (1995) |
| 2 | Moderate | S | c.53G>T | p.Cys18Phe | 0.317 | Tolerated | benign | 0.001 | benign | 0.002 | neutral | -47 | 1.04 | low | 361 | possibly damaging | -1.02 | Neutral | 0.321545 | benign | 0.324938 | bening | 0.23979 | bening | Centre B37 (unpublished) |
| <1 | Severe | S | c.59T>C | p.Leu20Ser | 0.044 | Damaging | benign | 0.047 | benign | 0.022 | neutral | -59 | 2.865 | medium | 176 | probably begign | -1.96 | Neutral | 0.334183 | benign | 0.35249 | bening | 0.50318 | damaging | Belvini et al (2005) |
| 40 | Mild | NS | c.62G>T | p.Gly21Val | 0.043 | Damaging | benign | 0.029 | benign | 0.01 | neutral | -51 | 2.075 | medium | 324 | possibly damaging | -1.3 | Neutral | 0.449942 | benign | 0.458223 | bening | 0.430054 | bening | Centre B31 (unpublished) |
| <1 | Severe | S | c.68T>C | p.Leu23Pro | 0.002 | Damaging | probably damaging | 0.985 | possibly damaging | 0.707 | effect | 54 | 2.74 | medium | 324 | possibly damaging | -2.58 | Deleterious | 0.816813 | damaging | 0.814203 | damaging | 0.734774 | damaging | Centre B14 (unpublished) |
| <1 | Severe | S | c.71T>C | p.Leu24Pro | 0.051 | Tolerated | benign | 0.022 | benign | 0.03 | neutral | -82 | 1.955 | medium | 176 | probably begign | -1.62 | Neutral | 0.343323 | benign | 0.362782 | bening | 0.517873 | damaging | Kwon et al (2008) |
| 2 | Moderate | S | c.77C>A | p.Ala26Asp | 0.035 | Damaging | benign | 0.048 | benign | 0.036 | neutral | -36 | 1.61 | low | 361 | possibly damaging | -1.85 | Neutral | 0.406417 | benign | 0.414767 | bening | 0.368021 | bening | Centre B25 (unpublished) |
| <5 | Moderate | S | c.82T>C | p.Cys28Arg | 0.527 | Tolerated | benign | 0.002 | benign | 0.005 | neutral | -14 | 2.08 | medium | 176 | probably begign | -0.6 | Neutral | 0.158274 | benign | 0.16627 | bening | 0.23735 | bening | Miller et al (2012) |
| <1 | Severe | S | c.82T>G | p.Cys28Gly | 0.497 | Tolerated | benign | 0 | benign | 0.001 | neutral | -51 | 1.385 | low | 176 | probably begign | -0.08 | Neutral | 0.160911 | benign | 0.170707 | bening | 0.243685 | bening | Liu et al (2000) |
| <1 | Severe | S | c.83G>A | p.Cys28Tyr | 0.043 | Damaging | benign | 0.267 | benign | 0.131 | effect | 6 | 2.08 | medium | 176 | probably begign | -1.49 | Neutral | 0.280441 | benign | 0.300536 | bening | 0.429015 | bening | Bicocchi et al (2006) |
| <1 | Severe | S | c.84T>G | p.Cys28Trp | 0.018 | Damaging | possibly damaging | 0.887 | possibly damaging | 0.662 | effect | 30 | 2.08 | medium | 176 | probably begign | -1.98 | Neutral | 0.402341 | benign | 0.431415 | bening | 0.615845 | damaging | Nielsen et al (1995) |
| <1 | Severe | S | c.86C>T | p.Thr29Ile | 0.011 | Damaging | benign | 0.053 | benign | 0.091 | neutral | -35 | 1.39 | low | 91 | probably begign | -2.04 | Neutral | 0.228185 | benign | 0.246825 | bening | 0.352344 | bening | Centre B21 (unpublished) |
| <1 | Severe | S | c.88G>A | p.Val30Ile | 0.009 | Damaging | probably damaging | 0.998 | possibly damaging | 0.882 | neutral | -7 | 3.025 | medium | 750 | probably damaging | -0.73 | Neutral | 0.61361 | damaging | 0.633964 | damaging | 0.680925 | damaging | Radic et al (2013) |
| <1 | Severe | S | c.88G>C | p.Val30Leu | 0.142 | Tolerated | probably damaging | 0.984 | possibly damaging | 0.728 | neutral | -45 | 2.33 | medium | 176 | probably begign | -2.2 | Neutral | 0.428345 | benign | 0.456841 | bening | 0.652142 | damaging | Giannel et al (1994) |
| <1 | Severe | S | c.109G>A | p.Ala37Thr | 0 | Damaging | probably damaging | 1 | probably damaging | 0.978 | effect | 34 | 3.025 | medium | 750 | probably damaging | -3.35 | Deleterious | 0.80056 | damaging | 0.797397 | damaging | 0.710784 | damaging | Chu et al (1996) |
| <1 | Severe | S | c.110C>T | p.Ala37Val | 0 | Damaging | probably damaging | 0.998 | probably damaging | 0.977 | effect | 11 | 3.025 | medium | 750 | probably damaging | -3.35 | Deleterious | 0.782977 | damaging | 0.779371 | damaging | 0.685051 | damaging | Oldenburg et al (1997) |
| <1 | Severe | S | c.110C>A | p.Ala37Asp | 0 | Damaging | probably damaging | 1 | probably damaging | 0.977 | effect | 67 | 3.025 | medium | 91 | probably begign | -5.03 | Deleterious | 0.689444 | damaging | 0.69733 | damaging | 0.791997 | damaging | Belvini et al (2005) |
| <1 | Severe | S | c.112A>C | p.Asn38His | 0.385 | Tolerated | benign | 0.012 | benign | 0.015 | neutral | -81 | -0.205 | low | 750 | probably damaging | -0.3 | Neutral | 0.380517 | benign | 0.384621 | bening | 0.324987 | bening | Quadros et al, (2009) |
| <1 | Severe | S | c.127C>T | p.Arg43Trp | 0 | Damaging | probably damaging | 1 | probably damaging | 0.999 | effect | 84 | 3.06 | medium | 750 | probably damaging | -6.35 | Deleterious | 0.900085 | damaging | 0.899288 | damaging | 0.856233 | damaging | Radic et al (2013) |
| <1 | Severe | S | c.128G>A | p.Arg43Gln | 0 | Damaging | probably damaging | 1 | probably damaging | 0.988 | effect | 68 | 3.06 | medium | 750 | probably damaging | -3.22 | Deleterious | 0.859552 | damaging | 0.857776 | damaging | 0.796974 | damaging | Radic et al (2013) |
| <1 | Severe | S | c.128G>T | p.Arg43Leu | 0 | Damaging | probably damaging | 1 | probably damaging | 0.991 | effect | 75 | 3.06 | medium | 176 | probably begign | -5.57 | Deleterious | 0.709568 | damaging | 0.717902 | damaging | 0.821365 | damaging | Kwon et al (2008) |
| <1 | Severe | S | c.135G>C | p.Lys45Asn | 0.003 | Damaging | probably damaging | 0.995 | possibly damaging | 0.873 | effect | 53 | 2.475 | medium | 176 | probably begign | -2.74 | Deleterious | 0.63933 | damaging | 0.646578 | damaging | 0.719549 | damaging | Belvini et al (2005) |
| <1 | Severe | S | c.135G>T | p.Lys45Asn | 0.003 | Damaging | probably damaging | 0.995 | possibly damaging | 0.873 | effect | 53 | 2.475 | medium | 750 | probably damaging | -2.74 | Deleterious | 0.805996 | damaging | 0.803538 | damaging | 0.719549 | damaging | Thompson et al (1992) |
| <1 | Severe | S | c.137G>A | p.Arg46Lys | 0 | Damaging | probably damaging | 1 | probably damaging | 0.983 | effect | 69 | 3.06 | medium | 750 | probably damaging | -2.52 | Deleterious | 0.861835 | damaging | 0.860114 | damaging | 0.800312 | damaging | Heit et al (1998) |
| <1 | Severe | S | c.137G>C | p.Arg46Thr | 0 | Damaging | probably damaging | 1 | probably damaging | 0.988 | effect | 71 | 3.06 | medium | 750 | probably damaging | -5.03 | Deleterious | 0.866502 | damaging | 0.864893 | damaging | 0.807135 | damaging | Centre B14 (unpublished) |
| 2 | Moderate | S | c.138G>T | p.Arg47Ser | 0 | Damaging | probably damaging | 1 | probably damaging | 0.988 | effect | 64 | 3.06 | medium | 750 | probably damaging | -5.03 | Deleterious | 0.850752 | damaging | 0.848763 | damaging | 0.784109 | damaging | Wulff et al (1999) |
| <1 | Severe | S | c.138G>C | p.Arg47Ser | 0 | Damaging | probably damaging | 1 | probably damaging | 0.988 | effect | 64 | 3.06 | medium | 750 | probably damaging | -5.03 | Deleterious | 0.850752 | damaging | 0.848763 | damaging | 0.784109 | damaging | Diuguid et al (1986) |
| 6 | Mild | NS | c.142A>G | p.Asn48Asp | 0 | Damaging | probably damaging | 0.999 | probably damaging | 0.95 | effect | 72 | 3.405 | medium | 750 | probably damaging | -4.1 | Deleterious | 0.879781 | damaging | 0.878133 | damaging | 0.826034 | damaging | Koeberl et al (1990) |
| 4 | Moderate | S | c.142A>T | p.Asn48Tyr | 0 | Damaging | probably damaging | 0.979 | possibly damaging | 0.472 | effect | 71 | 2.71 | medium | 750 | probably damaging | -6.38 | Deleterious | 0.850423 | damaging | 0.848634 | damaging | 0.783924 | damaging | Costa et al (2000) |
| 1 | Moderate | S | c.143A>T | p.Asn48Ile | 0 | Damaging | probably damaging | 1 | probably damaging | 0.942 | effect | 69 | 3.405 | medium | 750 | probably damaging | -6.78 | Deleterious | 0.872898 | damaging | 0.871092 | damaging | 0.815984 | damaging | Poon et al (1993) |
| 5 | Moderate | S | c.145T>C | p.Ser49Pro | 0.002 | Damaging | probably damaging | 0.965 | possibly damaging | 0.753 | effect | 85 | 2.985 | medium | 324 | possibly damaging | -3.17 | Deleterious | 0.894165 | damaging | 0.892958 | damaging | 0.847198 | damaging | Montejo et al (1999) |
| 23 | Mild | NS | c.148G>A | p.Gly50Ser | 0.259 | Tolerated | probably damaging | 0.989 | possibly damaging | 0.904 | neutral | -1 | 2.49 | medium | 456 | probably damaging | -1.68 | Neutral | 0.549489 | damaging | 0.565382 | damaging | 0.583024 | damaging | Van de Water et al (1996) |
| 7 | Mild | NS | c.151A>G | p.Lys51Glu | 0.296 | Tolerated | benign | 0.282 | benign | 0.197 | effect | 25 | 0.69 | neutral | 220 | possibly damaging | -0.86 | Neutral | 0.341417 | benign | 0.348288 | bening | 0.273123 | bening | Koeberl et al (1990) |
| <1 | Severe | S | c.155T>C | p.Leu52Ser | 0.002 | Damaging | possibly damaging | 0.949 | probably damaging | 0.995 | effect | 59 | 3.19 | medium | 361 | possibly damaging | -3.42 | Deleterious | 0.835977 | damaging | 0.832999 | damaging | 0.761605 | damaging | Belvini et al (2005) |
| <1 | Severe | S | c.157G>A | p.Glu53Lys | 0 | Damaging | probably damaging | 1 | probably damaging | 0.997 | effect | 69 | 3.64 | high | 750 | probably damaging | -3.39 | Deleterious | 0.879809 | damaging | 0.877951 | damaging | 0.825775 | damaging | Costa et al (2000) |
| 5 | Moderate | S | c.158A>C | p.Glu53Ala | 0 | Damaging | probably damaging | 0.999 | probably damaging | 0.989 | effect | 42 | 3.64 | high | 750 | probably damaging | -5.08 | Deleterious | 0.829692 | damaging | 0.826614 | damaging | 0.752492 | damaging | Winship and Dragon (1991) |
| 2 | Moderate | S | c.158A>G | p.Glu53Gly | 0 | Damaging | probably damaging | 0.999 | probably damaging | 0.997 | effect | 67 | 3.64 | high | 750 | probably damaging | -5.93 | Deleterious | 0.875109 | damaging | 0.873128 | damaging | 0.81889 | damaging | Belvini et al (2005) |
| <1 | Severe | S | c.158A>T | p.Glu53Val | 0 | Damaging | probably damaging | 1 | probably damaging | 0.99 | effect | 52 | 3.64 | high | 750 | probably damaging | -5.93 | Deleterious | 0.845526 | damaging | 0.842839 | damaging | 0.775653 | damaging | Centre B31 (unpublished) |
| 2 | Moderate | S | c.160G>A | p.Glu54Lys | 0 | Damaging | probably damaging | 0.999 | probably damaging | 0.968 | effect | 70 | 3.58 | high | 750 | probably damaging | -3.39 | Deleterious | 0.880238 | damaging | 0.878436 | damaging | 0.826466 | damaging | Centre B14 (unpublished) |
| <1 | Severe | S | c.160G>C | p.Glu54Gln | 0 | Damaging | probably damaging | 0.997 | probably damaging | 0.99 | effect | 46 | 3.58 | high | 750 | probably damaging | -2.54 | Deleterious | 0.833505 | damaging | 0.830555 | damaging | 0.758117 | damaging | Radic et al (2013) |
| 4 | Moderate | S | c.161A>C | p.Glu54Ala | 0 | Damaging | probably damaging | 0.998 | probably damaging | 0.996 | effect | 41 | 3.03 | medium | 750 | probably damaging | -5.08 | Deleterious | 0.809148 | damaging | 0.806168 | damaging | 0.723305 | damaging | Saad et al (1994) |
| 2 | Moderate | S | c.161A>G | p.Glu54Gly | 0 | Damaging | probably damaging | 0.999 | probably damaging | 0.97 | effect | 47 | 3.58 | high | 750 | probably damaging | -5.93 | Deleterious | 0.835388 | damaging | 0.832502 | damaging | 0.760896 | damaging | Bottema et al (1991) |
| <1 | Severe | S | c.162G>C | p.Glu54Asp | 0 | Damaging | probably damaging | 0.993 | benign | 0.01 | effect | 49 | 3.58 | high | 750 | probably damaging | -2.54 | Deleterious | 0.837588 | damaging | 0.8347 | damaging | 0.764034 | damaging | Yu et al (2012) |
| 15 | Mild | NS | c.163T>A | p.Phe55Ile | 0.129 | Tolerated | benign | 0.016 | possibly damaging | 0.517 | neutral | -40 | 0.59 | neutral | 324 | possibly damaging | -1.54 | Neutral | 0.341167 | benign | 0.34791 | bening | 0.272583 | bening | Bicocchi et al (2006) |
| <1 | Severe | S | c.164T>C | p.Phe55Ser | 0.003 | Damaging | possibly damaging | 0.7 | probably damaging | 0.909 | effect | 66 | 1.375 | low | 324 | possibly damaging | -3.98 | Deleterious | 0.721744 | damaging | 0.716479 | damaging | 0.595274 | damaging | Ketterling et al (1999) |
| <1 | Severe | S | c.164T>G | p.Phe55Cys | 0.003 | Damaging | probably damaging | 0.995 | benign | 0.441 | effect | 30 | 2.18 | medium | 324 | possibly damaging | -3.57 | Deleterious | 0.761037 | damaging | 0.75791 | damaging | 0.654416 | damaging | Montejo et al (1999) |
| <5 | Moderate | S | c.164T>A | p.Phe55Tyr | 0.004 | Damaging | possibly damaging | 0.602 | benign | 0.068 | effect | 47 | 2.18 | medium | 324 | possibly damaging | -1.5 | Neutral | 0.55052 | damaging | 0.566865 | damaging | 0.585142 | damaging | (Centers for Disease Control and Prevention, 2018) |
| <1 | Severe | S | c.172G>A | p.Gly58Arg | 0.001 | Damaging | probably damaging | 1 | probably damaging | 0.999 | effect | 59 | 3.6 | high | 750 | probably damaging | -6.08 | Deleterious | 0.857165 | damaging | 0.854782 | damaging | 0.792701 | damaging | Giannelli et al (1993) |
| <1 | Severe | S | c.172G>C | p.Gly58Arg | 0.001 | Damaging | probably damaging | 1 | probably damaging | 0.998 | effect | 59 | 3.6 | high | 750 | probably damaging | -6.08 | Deleterious | 0.857165 | damaging | 0.854782 | damaging | 0.792701 | damaging | Kwon et al (2008) |
| 25 | Mild | NS | c.173G>A | p.Gly58Glu | 0.002 | Damaging | probably damaging | 1 | probably damaging | 0.984 | neutral | -87 | 3.6 | high | 750 | probably damaging | -5.81 | Deleterious | 0.925131 | damaging | 0.924377 | damaging | 0.892048 | damaging | Costa et al (2000) |
| <5 | Moderate | S | c.173G>C | p.Gly58Ala | 0.033 | Damaging | probably damaging | 1 | probably damaging | 1 | effect | 35 | 2.795 | medium | 750 | probably damaging | -4.42 | Deleterious | 0.788021 | damaging | 0.784357 | damaging | 0.69217 | damaging | Belvini et al (2005) |
| <1 | Severe | S | c.173G>T | p.Gly58Val | 0.001 | Damaging | probably damaging | 1 | probably damaging | 0.987 | effect | 55 | 3.6 | high | 750 | probably damaging | -6.86 | Deleterious | 0.849565 | damaging | 0.846998 | damaging | 0.78159 | damaging | Jayandharan et al (2005) |
| >5 | Mild | NS | c.178C>T | p.Leu60Phe | 0.029 | Damaging | probably damaging | 1 | probably damaging | 1 | effect | 43 | 2.6 | medium | 750 | probably damaging | -3.24 | Deleterious | 0.791599 | damaging | 0.788315 | damaging | 0.697819 | damaging | Miller et al (2012) |
| 6 | Mild | NS | c.179T>C | p.Leu60Pro | 0.001 | Damaging | probably damaging | 1 | probably damaging | 1 | effect | 73 | 3.15 | medium | 750 | probably damaging | -5.75 | Deleterious | 0.874137 | damaging | 0.872604 | damaging | 0.818141 | damaging | Centre B11 (unpublished) |
| 1 | Moderate | S | c.187G>A | p.Glu63Lys | 0 | Damaging | probably damaging | 1 | probably damaging | 1 | effect | 63 | 3.645 | high | 750 | probably damaging | -3.39 | Deleterious | 0.866751 | damaging | 0.864573 | damaging | 0.806678 | damaging | Tartary et al (1993) |
| <1 | Severe | S | c.188A>G | p.Glu63Gly | 0 | Damaging | probably damaging | 1 | probably damaging | 0.999 | effect | 65 | 3.645 | high | 750 | probably damaging | -5.93 | Deleterious | 0.871018 | damaging | 0.868943 | damaging | 0.812915 | damaging | Yu et al (2012) |
| <1 | Severe | S | c.188A>T | p.Glu63Val | 0 | Damaging | probably damaging | 1 | probably damaging | 1 | effect | 48 | 3.3 | medium | 750 | probably damaging | -5.93 | Deleterious | 0.828704 | damaging | 0.825934 | damaging | 0.751521 | damaging | Jayandharan et al (2005) |
| <1 | Severe | S | c.190T>C | p.Cys64Arg | 0 | Damaging | probably damaging | 1 | probably damaging | 1 | effect | 79 | 3.65 | high | 750 | probably damaging | -10.17 | Deleterious | 0.90476 | damaging | 0.903495 | damaging | 0.862239 | damaging | Enayat et al (2004) |
| <1 | Severe | S | c.191G>A | p.Cys64Tyr | 0 | Damaging | probably damaging | 1 | probably damaging | 1 | effect | 73 | 3.65 | high | 750 | probably damaging | -9.32 | Deleterious | 0.88956 | damaging | 0.887928 | damaging | 0.840017 | damaging | Mahajan et al (2004) |
| <5 | Moderate | S | c.197A>T | p.Glu66Val | 0 | Damaging | probably damaging | 1 | probably damaging | 1 | effect | 38 | 3.645 | high | 750 | probably damaging | -5.93 | Deleterious | 0.824668 | damaging | 0.821473 | damaging | 0.745152 | damaging | Wulff et al (1995) |
| >5 | Mild | NS | c.196G>A | p.Glu66Lys | 0 | Damaging | probably damaging | 1 | probably damaging | 0.993 | effect | 52 | 3.3 | medium | 750 | probably damaging | -3.39 | Deleterious | 0.835371 | damaging | 0.832762 | damaging | 0.761267 | damaging | (Centers for Disease Control and Prevention, 2018) |
| 1 | Moderate | S | c.199G>A | p.Glu67Lys | 0 | Damaging | probably damaging | 0.999 | probably damaging | 0.997 | effect | 71 | 3.645 | high | 750 | probably damaging | -3.39 | Deleterious | 0.884451 | damaging | 0.882691 | damaging | 0.832542 | damaging | Sommer et al (1995) |
| >5 | Mild | NS | c.201A>C | p.Glu67Asp | 0 | Damaging | probably damaging | 0.999 | probably damaging | 1 | effect | 47 | 3.645 | high | 750 | probably damaging | -2.54 | Deleterious | 0.837251 | damaging | 0.834351 | damaging | 0.763536 | damaging | Rydz et al (2013) |
| <1 | Severe | S | c.205T>C | p.Cys69Arg | 0 | Damaging | probably damaging | 1 | probably damaging | 1 | effect | 83 | 3.65 | high | 750 | probably damaging | -10.17 | Deleterious | 0.91556 | damaging | 0.914556 | damaging | 0.878029 | damaging | Bicocchi et al (2006) |
| <1 | Severe | S | c.205T>G | p.Cys69Gly | 0 | Damaging | probably damaging | 1 | probably damaging | 0.999 | effect | 83 | 3.65 | high | 750 | probably damaging | -10.17 | Deleterious | 0.91556 | damaging | 0.914556 | damaging | 0.878029 | damaging | Yu et al (2012) |
| <1 | Severe | S | c.206G>A | p.Cys69Tyr | 0 | Damaging | probably damaging | 1 | probably damaging | 0.946 | effect | 66 | 3.65 | high | 750 | probably damaging | -9.32 | Deleterious | 0.873343 | damaging | 0.87132 | damaging | 0.816309 | damaging | Yu et al (2012) |
| 2 | Moderate | S | c.212T>C | p.Phe71Ser | 0.031 | Damaging | probably damaging | 0.997 | probably damaging | 0.986 | effect | 26 | 2.565 | medium | 220 | possibly damaging | -4.12 | Deleterious | 0.769812 | damaging | 0.765991 | damaging | 0.665952 | damaging | Ludwig et al (1992) |
| 10 | Mild | NS | c.212T>G | p.Phe71Cys | 0.014 | Damaging | probably damaging | 1 | probably damaging | 0.993 | effect | 48 | 2.565 | medium | 220 | possibly damaging | -4.1 | Deleterious | 0.800279 | damaging | 0.797457 | damaging | 0.71087 | damaging | Belvini et al (2005) |
| <1 | Severe | S | c.214G>C | p.Glu72Gln | 0 | Damaging | probably damaging | 1 | probably damaging | 1 | effect | 37 | 3.625 | high | 750 | probably damaging | -2.54 | Deleterious | 0.822848 | damaging | 0.819627 | damaging | 0.742518 | damaging | Ivaskevicius et al (2001) |
| <1 | Severe | S | c.215A>G | p.Glu72Gly | 0 | Damaging | probably damaging | 1 | probably damaging | 0.999 | effect | 64 | 3.625 | high | 750 | probably damaging | -5.92 | Deleterious | 0.868298 | damaging | 0.866175 | damaging | 0.808965 | damaging | Liu et al (2000) |
| <1 | Severe | S | c.217G>A | p.Glu73Lys | 0 | Damaging | probably damaging | 1 | probably damaging | 0.999 | effect | 72 | 3.645 | high | 750 | probably damaging | -3.39 | Deleterious | 0.887001 | damaging | 0.885312 | damaging | 0.836283 | damaging | Chen et al (1989b) |
| 7 | Mild | NS | c.218A>C | p.Glu73Ala | 0 | Damaging | probably damaging | 1 | probably damaging | 0.997 | effect | 46 | 3.645 | high | 750 | probably damaging | -5.08 | Deleterious | 0.835868 | damaging | 0.832944 | damaging | 0.761527 | damaging | Attali et al (1999) |
| <1 | Severe | S | c.218A>T | p.Glu73Val | 0 | Damaging | probably damaging | 1 | probably damaging | 0.998 | effect | 58 | 3.645 | high | 750 | probably damaging | -5.93 | Deleterious | 0.856668 | damaging | 0.854246 | damaging | 0.791936 | damaging | Wang et al (1990) |
| <1 | Severe | S | c.219A>C | p.Glu73Asp | 0 | Damaging | probably damaging | 1 | probably damaging | 0.998 | effect | 43 | 3.645 | high | 750 | probably damaging | -2.54 | Deleterious | 0.831418 | damaging | 0.828386 | damaging | 0.755021 | damaging | Yu et al (2012) |
| <1 | Severe | S | c.219A>T | p.Glu73Asp | 0 | Damaging | probably damaging | 1 | probably damaging | 0.997 | effect | 43 | 3.645 | high | 750 | probably damaging | -2.54 | Deleterious | 0.831418 | damaging | 0.828386 | damaging | 0.755021 | damaging | Costa et al (2000) |
| >5 | Mild | NS | c.224G>A | p.Arg75Gln | 0.003 | Damaging | probably damaging | 1 | probably damaging | 1 | effect | 62 | 3.645 | high | 750 | probably damaging | -3.08 | Deleterious | 0.864168 | damaging | 0.861886 | damaging | 0.802841 | damaging | Miller et al (2012) |
| 1 | Moderate | S | c.224G>C | p.Arg75Pro | 0.001 | Damaging | probably damaging | 1 | probably damaging | 0.999 | effect | 78 | 2.665 | medium | 750 | probably damaging | -5.54 | Deleterious | 0.869406 | damaging | 0.868309 | damaging | 0.812011 | damaging | Thorland et al (1995) |
| >5 | Mild | NS | c.223C>G | p.Arg75Gly | 0.001 | Damaging | probably damaging | 1 | probably damaging | 0.99 | effect | 71 | 3.215 | medium | 750 | probably damaging | -5.59 | Deleterious | 0.871452 | damaging | 0.869787 | damaging | 0.81412 | damaging | Konkle et al (2014) |
| <5 | Moderate | S | c.226G>A | p.Glu76Lys | 0 | Damaging | probably damaging | 0.999 | probably damaging | 0.999 | effect | 73 | 3.215 | medium | 750 | probably damaging | -3.39 | Deleterious | 0.876252 | damaging | 0.874707 | damaging | 0.821144 | damaging | Belvini et al (2005) |
| <5 | Moderate | S | c.226G>C | p.Glu76Gln | 0 | Damaging | probably damaging | 1 | probably damaging | 0.997 | effect | 54 | 3.645 | high | 750 | probably damaging | -2.54 | Deleterious | 0.849201 | damaging | 0.846599 | damaging | 0.78102 | damaging | Belvini et al (2005) |
| <1 | Severe | S | c.227A>C | p.Glu76Ala | 0 | Damaging | probably damaging | 0.999 | probably damaging | 0.997 | effect | 47 | 3.3 | medium | 750 | probably damaging | -5.08 | Deleterious | 0.826954 | damaging | 0.824133 | damaging | 0.748949 | damaging | Rydz et al (2013) |
| <5 | Moderate | S | c.228A>C | p.Glu76Asp | 0 | Damaging | probably damaging | 1 | probably damaging | 1 | effect | 42 | 3.645 | high | 750 | probably damaging | -2.54 | Deleterious | 0.830001 | damaging | 0.826935 | damaging | 0.75295 | damaging | Yu et al (2012) |
| <5 | Moderate | S | c.233T>C | p.Phe78Ser | 0 | Damaging | probably damaging | 1 | possibly damaging | 0.871 | effect | 74 | 3.17 | medium | 750 | probably damaging | -6.77 | Deleterious | 0.877409 | damaging | 0.875948 | damaging | 0.822915 | damaging | Centre B31 (unpublished) |
| <1 | Severe | S | c.235G>A | p.Glu79Lys | 0 | Damaging | probably damaging | 0.986 | possibly damaging | 0.881 | effect | 45 | 2.19 | medium | 456 | probably damaging | -2.25 | Neutral | 0.612594 | damaging | 0.634002 | damaging | 0.680979 | damaging | Belvini et al (2005) |
| <1 | Severe | S | c.235G>C | p.Glu79Gln | 0 | Damaging | probably damaging | 0.98 | possibly damaging | 0.907 | effect | 47 | 2 | medium | 456 | probably damaging | -1.92 | Neutral | 0.605263 | damaging | 0.626737 | damaging | 0.670608 | damaging | Belvini et al (2005) |
| <5 | Moderate | S | c.236A>C | p.Glu79Ala | 0 | Damaging | probably damaging | 0.973 | probably damaging | 0.918 | effect | 20 | 3.125 | medium | 456 | probably damaging | -4.43 | Deleterious | 0.786828 | damaging | 0.782979 | damaging | 0.690201 | damaging | Yu et al (2012) |
| 1 | Moderate | S | c.236A>G | p.Glu79Gly | 0 | Damaging | probably damaging | 0.96 | probably damaging | 0.962 | effect | 48 | 2.78 | medium | 456 | probably damaging | -5.27 | Deleterious | 0.804281 | damaging | 0.801118 | damaging | 0.716096 | damaging | Wulff et al (1998) |
| 4 | Moderate | S | c.237A>C | p.Glu79Asp | 0 | Damaging | probably damaging | 0.994 | probably damaging | 0.987 | effect | 45 | 2.78 | medium | 456 | probably damaging | -2.37 | Neutral | 0.638631 | damaging | 0.659955 | damaging | 0.718028 | damaging | Koeberl et al (1989) |
| <1 | Severe | S | c.236A>T | p.Glu79Val | 0 | Damaging | probably damaging | 0.997 | possibly damaging | 0.903 | effect | 25 | 2.67 | medium | 456 | probably damaging | -5.29 | Deleterious | 0.778283 | damaging | 0.774965 | damaging | 0.678762 | damaging | Rydz et al (2013) |
| <1 | Severe | S | c.251C>G | p.Thr84Arg | 0.001 | Damaging | probably damaging | 1 | probably damaging | 0.999 | effect | 80 | 3.58 | high | 750 | probably damaging | -4.87 | Deleterious | 0.905238 | damaging | 0.904035 | damaging | 0.863009 | damaging | Centre B24 (unpublished) |
| >5 | Mild | NS | c.251C>T | p.Thr84Ile | 0.001 | Damaging | probably damaging | 1 | probably damaging | 0.997 | effect | 75 | 3.58 | high | 750 | probably damaging | -4.88 | Deleterious | 0.892321 | damaging | 0.890806 | damaging | 0.844125 | damaging | Belvini et al (2005) |
| <1 | Severe | S | c.259T>G | p.Phe87Val | 0.001 | Damaging | probably damaging | 0.999 | probably damaging | 1 | effect | 71 | 3.585 | high | 750 | probably damaging | -5.77 | Deleterious | 0.882566 | damaging | 0.880801 | damaging | 0.829843 | damaging | Bicocchi et al (2006) |
| <5 | Moderate | S | c.260T>G | p.Phe87Cys | 0 | Damaging | probably damaging | 1 | probably damaging | 0.999 | effect | 53 | 3.585 | high | 750 | probably damaging | -6.62 | Deleterious | 0.845699 | damaging | 0.843067 | damaging | 0.775978 | damaging | Belvini et al (2005) |
| <1 | Severe | S | c.262T>C | p.Trp88Arg | 0 | Damaging | probably damaging | 1 | benign | 0.126 | effect | 90 | 3.565 | high | 750 | probably damaging | -11.96 | Deleterious | 0.933303 | damaging | 0.932806 | damaging | 0.90408 | damaging | Mahajan et al (2007) |
| 14 | Mild | NS | c.265A>G | p.Lys89Glu | 0.282 | Tolerated | benign | 0.276 | probably damaging | 1 | effect | 9 | 1.265 | low | 362 | possibly damaging | -1.07 | Neutral | 0.358026 | benign | 0.364665 | bening | 0.2965 | bening | Centre B28 (unpublished) |
| <1 | Severe | S | c.271T>A | p.Tyr91Asn | 0 | Damaging | probably damaging | 1 | probably damaging | 1 | effect | 86 | 3.455 | medium | 750 | probably damaging | -7.67 | Deleterious | 0.918324 | damaging | 0.917568 | damaging | 0.882328 | damaging | Ghosh et al (2009) |
| 9 | Mild | NS | c.271T>G | p.Tyr91Asp | 0 | Damaging | probably damaging | 1 | probably damaging | 1 | effect | 89 | 3.455 | medium | 750 | probably damaging | -8.51 | Deleterious | 0.927074 | damaging | 0.926529 | damaging | 0.895121 | damaging | Gostout et al (1993) |
| <5 | Moderate | S | c.272A>G | p.Tyr91Cys | 0 | Damaging | probably damaging | 1 | benign | 0.073 | effect | 70 | 3.455 | medium | 750 | probably damaging | -7.66 | Deleterious | 0.876724 | damaging | 0.874963 | damaging | 0.82151 | damaging | Li et al (2000) |
| >5 | Mild | NS | c.398C>T | p.Thr133Ile | 0.484 | Tolerated | benign | 0 | benign | 0.001 | neutral | -45 | 1.295 | LOW | 176 | probably begign | -0.32 | Neutral | 0.735359 | damaging | 0.731429 | damaging | 0.616614 | damaging | Rydz et al (2013) |
| <1 | Severe | S | c.400T>C | p.Cys134Arg | 0 | Damaging | probably damaging | 1 | probably damaging | 1 | effect | 59 | 4.23 | HIGH | 750 | probably damaging | -7.52 | Deleterious | 0.771228 | damaging | 0.767707 | damaging | 0.668401 | damaging | Belvini et al (2005) |
| <1 | Severe | S | c.401G>C | p.Cys134Ser | 0 | Damaging | probably damaging | 1 | probably damaging | 1 | effect | 60 | 3.88 | HIGH | 750 | probably damaging | -6.94 | Deleterious | 0.671185 | damaging | 0.663607 | damaging | 0.519798 | damaging | Belvini et al (2005) |
| <1 | Severe | S | c.400T>G | p.Cys134Gly | 0 | Damaging | probably damaging | 1 | probably damaging | 1 | effect | 66 | 4.23 | HIGH | 750 | probably damaging | -8.3 | Deleterious | 0.719183 | damaging | 0.71363 | damaging | 0.591207 | damaging | (Centers for Disease Control and Prevention, 2018) |
| <1 | Severe | S | c.401G>A | p.Cys134Tyr | 0 | Damaging | probably damaging | 1 | probably damaging | 1 | effect | 66 | 4.23 | HIGH | 750 | probably damaging | -8.44 | Deleterious | 0.78287 | damaging | 0.782587 | damaging | 0.689642 | damaging | Tartary et al (1990) |
| 15 | Mild | NS | c.407T>C | p.Ile136Thr | 0.184 | Tolerated | benign | 0.007 | benign | 0.018 | neutral | -41 | 0.5 | NEUTRAL | 324 | possibly damaging | -1.59 | Neutral | 0.729733 | damaging | 0.728444 | damaging | 0.612353 | damaging | Saad et al (1994) |
| >5 | Mild | NS | c.412A>C | p.Asn138His | 0 | Damaging | probably damaging | 1 | probably damaging | 0.958 | effect | 43 | 3.09 | MEDIUM | 750 | probably damaging | -4.32 | Deleterious | 0.620173 | damaging | 0.626463 | damaging | 0.690834 | damaging | Belvini et al (2005) |
| <1 | Severe | S | c.413A>G | p.Asn138Ser | 0 | Damaging | possibly damaging | 0.905 | benign | 0.221 | effect | 27 | 2.845 | MEDIUM | 750 | probably damaging | -4.32 | Deleterious | 0.645071 | damaging | 0.651585 | damaging | 0.726696 | damaging | Yu et al (2012) |
| <1 | Severe | S | c.414T>A | p.Asn138Lys | 0 | Damaging | probably damaging | 0.997 | possibly damaging | 0.749 | effect | 55 | 3.03 | MEDIUM | 750 | probably damaging | -5.19 | Deleterious | 0.678917 | damaging | 0.675276 | damaging | 0.536455 | damaging | Attali et al (1999) |
| <1 | Severe | S | c.415G>A | p.Gly139Ser | 0 | Damaging | probably damaging | 0.996 | probably damaging | 0.983 | effect | 37 | 3.105 | MEDIUM | 750 | probably damaging | -5.19 | Deleterious | 0.551017 | damaging | 0.536902 | damaging | 0.338927 | bening | Balraj et al (2012) |
| <1 | Severe | S | c.416G>A | p.Gly139Asp | 0 | Damaging | probably damaging | 1 | probably damaging | 0.983 | effect | 62 | 2.395 | MEDIUM | 750 | probably damaging | -6.05 | Deleterious | 0.615583 | damaging | 0.608761 | damaging | 0.441506 | bening | Yu et al (2012) |
| <5 | Moderate | S | c.420A>T | p.Arg140Ser | 0.352 | Tolerated | benign | 0.05 | benign | 0.022 | effect | 27 | 1.24 | LOW | 176 | probably begign | 0.71 | Neutral | 0.615583 | damaging | 0.608761 | damaging | 0.441506 | bening | Miller et al (2012) |
| <1 | Severe | S | c.421T>C | p.Cys141Arg | 0 | Damaging | probably damaging | 1 | probably damaging | 1 | effect | 88 | 4.25 | HIGH | 750 | probably damaging | -10.38 | Deleterious | 0.951397 | damaging | 0.950389 | damaging | 0.92918 | damaging | Bicocchi et al (2006) |
| <1 | Severe | S | c.421T>A | p.Cys141Ser | 0 | Damaging | probably damaging | 1 | probably damaging | 0.999 | effect | 85 | 3.9 | HIGH | 750 | probably damaging | -8.65 | Deleterious | 0.921731 | damaging | 0.920253 | damaging | 0.886161 | damaging | Centre B22 (unpublished) |
| <1 | Severe | S | c.422G>A | p.Cys141Tyr | 0 | Damaging | probably damaging | 1 | probably damaging | 0.999 | effect | 89 | 4.25 | HIGH | 750 | probably damaging | -9.51 | Deleterious | 0.916947 | damaging | 0.915106 | damaging | 0.878814 | damaging | Radic et al (2013) |
| <1 | Severe | S | c.422G>C | p.Cys141Ser | 0 | Damaging | probably damaging | 1 | probably damaging | 0.999 | effect | 85 | 3.9 | HIGH | 750 | probably damaging | -8.65 | Deleterious | 0.828245 | damaging | 0.825237 | damaging | 0.750526 | damaging | Centre B14 (unpublished) |
| <1 | Severe | S | c.423C>G | p.Cys141Trp | 0 | Damaging | probably damaging | 1 | probably damaging | 1 | effect | 89 | 4.25 | HIGH | 750 | probably damaging | -9.51 | Deleterious | 0.807785 | damaging | 0.804431 | damaging | 0.720824 | damaging | Ljung et al (2001) |
| <1 | Severe | S | c.424G>A | p.Glu142Lys | 0.516 | Tolerated | benign | 0.014 | benign | 0.005 | neutral | -28 | 0.74 | NEUTRAL | 30 | probably begign | -1.46 | Neutral | 0.829555 | damaging | 0.826537 | damaging | 0.752381 | damaging | Enayat et al (2004) |
| <1 | Severe | S | c.427C>G | p.Gln143Glu | 0.011 | Damaging | probably damaging | 0.985 | possibly damaging | 0.805 | effect | 26 | 2.85 | MEDIUM | 176 | probably begign | -1.44 | Neutral | 0.821574 | damaging | 0.81785 | damaging | 0.73998 | damaging | Yu et al (2012) |
| <1 | Severe | S | c.427C>A | p.Gln143Lys | 0.018 | Damaging | probably damaging | 0.991 | possibly damaging | 0.828 | effect | 24 | 3.545 | HIGH | 176 | probably begign | -2.31 | Neutral | 0.817845 | damaging | 0.814586 | damaging | 0.735321 | damaging | Kwon et al (2008) |
| <1 | Severe | S | c.428A>G | p.Gln143Arg | 0.015 | Damaging | possibly damaging | 0.617 | benign | 0.373 | effect | 13 | 2.85 | MEDIUM | 176 | probably begign | -1.93 | Neutral | 0.802118 | damaging | 0.798521 | damaging | 0.712389 | damaging | Quadros et al, (2009) |
| <1 | Severe | S | c.433T>C | p.Cys145Arg | 0 | Damaging | probably damaging | 1 | probably damaging | 1 | effect | 79 | 4.25 | HIGH | 911 | probably damaging | -10.38 | Deleterious | 0.872031 | damaging | 0.869312 | damaging | 0.813443 | damaging | Koeberl et al (1990) |
| <1 | Severe | S | c.434G>A | p.Cys145Tyr | 0 | Damaging | probably damaging | 1 | probably damaging | 0.999 | effect | 76 | 4.25 | HIGH | 911 | probably damaging | -9.46 | Deleterious | 0.893814 | damaging | 0.891776 | damaging | 0.84551 | damaging | Yu et al (2012) |
| <1 | Severe | S | c.457G>A | p.Val153Met | 0.004 | Damaging | possibly damaging | 0.825 | possibly damaging | 0.61 | effect | 37 | 2.335 | MEDIUM | 176 | probably begign | -0.98 | Neutral | 0.883703 | damaging | 0.881531 | damaging | 0.830885 | damaging | Belvini et al (2005) |
| 20 | Mild | NS | c.461T>C | p.Val154Ala | 0.079 | Tolerated | benign | 0.031 | benign | 0.017 | effect | 27 | 1.72 | LOW | 220 | possibly damaging | -1.51 | Neutral | 0.872031 | damaging | 0.869312 | damaging | 0.813443 | damaging | Chen et al (1991a) |
| <1 | Severe | S | c.464G>A | p.Cys155Tyr | 0 | Damaging | probably damaging | 1 | probably damaging | 1 | effect | 86 | 4.25 | HIGH | 750 | probably damaging | -9.33 | Deleterious | 0.895471 | damaging | 0.893074 | damaging | 0.847363 | damaging | Attali et al (1999) |
| <1 | Severe | S | c.464G>C | p.Cys155Ser | 0 | Damaging | probably damaging | 1 | probably damaging | 1 | effect | 87 | 4.25 | HIGH | 750 | probably damaging | -8.6 | Deleterious | 0.838393 | damaging | 0.835078 | damaging | 0.764573 | damaging | Belvini et al (2005) |
| <1 | Severe | S | c.464G>T | p.Cys155Phe | 0 | Damaging | probably damaging | 1 | probably damaging | 1 | effect | 88 | 4.25 | HIGH | 750 | probably damaging | -9.46 | Deleterious | 0.779056 | damaging | 0.77387 | damaging | 0.677199 | damaging | Kwon et al (2008) |
| <1 | Severe | S | c.466T>C | p.Ser156Pro | 0.005 | Damaging | probably damaging | 0.999 | probably damaging | 0.909 | effect | 62 | 3.705 | HIGH | 750 | probably damaging | -3.31 | Deleterious | 0.852272 | damaging | 0.848767 | damaging | 0.784115 | damaging | Elmahmoudi et al (2011) |
| <1 | Severe | S | c.469T>C | p.Cys157Arg | 0.001 | Damaging | probably damaging | 1 | probably damaging | 0.999 | effect | 79 | 4.245 | HIGH | 911 | probably damaging | -10.2 | Deleterious | 0.581744 | damaging | 0.562871 | damaging | 0.375998 | bening | Lin and Shen (1993) |
| 2 | Moderate | S | c.470G>A | p.Cys157Tyr | 0.001 | Damaging | probably damaging | 1 | probably damaging | 0.999 | effect | 71 | 4.245 | HIGH | 911 | probably damaging | -9.3 | Deleterious | 0.771739 | damaging | 0.768465 | damaging | 0.669483 | damaging | Jaloma-Cruz et al (2000) |
| <5 | Moderate | S | c.470G>C | p.Cys157Ser | 0.002 | Damaging | probably damaging | 1 | probably damaging | 0.998 | effect | 74 | 4.245 | HIGH | 911 | probably damaging | -8.47 | Deleterious | 0.837762 | damaging | 0.834618 | damaging | 0.763917 | damaging | Jaloma-Cruz et al (2000) |
| <1 | Severe | S | c.478G>A | p.Gly160Arg | 0.001 | Damaging | probably damaging | 1 | probably damaging | 0.998 | effect | 75 | 3.865 | HIGH | 750 | probably damaging | -6.43 | Deleterious | 0.852611 | damaging | 0.849399 | damaging | 0.785016 | damaging | Jenkins et al (2008) |
| >5 | Mild | NS | c.479G>A | p.Gly160Glu | 0.002 | Damaging | probably damaging | 1 | probably damaging | 0.995 | effect | 76 | 3.315 | MEDIUM | 750 | probably damaging | -6.22 | Deleterious | 0.895163 | damaging | 0.893242 | damaging | 0.847602 | damaging | Wulff et al (1995) |
| 5 | Moderate | S | c.479G>C | p.Gly160Ala | 0.004 | Damaging | probably damaging | 1 | probably damaging | 0.976 | effect | 53 | 3.865 | HIGH | 750 | probably damaging | -4.78 | Deleterious | 0.837762 | damaging | 0.834618 | damaging | 0.763917 | damaging | Winship and Dragon (1991) |
| 1 | Moderate | S | c.479G>T | p.Gly160Val | 0.001 | Damaging | probably damaging | 1 | probably damaging | 0.995 | effect | 54 | 4.21 | HIGH | 750 | probably damaging | -7.16 | Deleterious | 0.956115 | damaging | 0.955162 | damaging | 0.935994 | damaging | Mukherjee et al (2004) |
| 4 | Moderate | S | c.482A>G | p.Tyr161Cys | 0.001 | Damaging | probably damaging | 1 | probably damaging | 1 | effect | 65 | 4.055 | HIGH | 750 | probably damaging | -7.54 | Deleterious | 0.931658 | damaging | 0.930357 | damaging | 0.900585 | damaging | Liu et al (2000) |
| <1 | Severe | S | c.487C>T | p.Leu163Phe | 0.004 | Damaging | probably damaging | 1 | probably damaging | 0.995 | effect | 55 | 1.72 | LOW | 750 | probably damaging | -3.32 | Deleterious | 0.950382 | damaging | 0.949291 | damaging | 0.927612 | damaging | Belvini et al (2005) |
| 25 | Mild | NS | c.491C>T | p.Ala164Val | 0.014 | Damaging | probably damaging | 0.972 | benign | 0.425 | neutral | -55 | 4.25 | HIGH | 324 | possibly damaging | -2.11 | Neutral | 0.959032 | damaging | 0.958149 | damaging | 0.940258 | damaging | Centre B26 (unpublished) |
| <1 | Severe | S | c.496A>T | p.Asn166Tyr | 0.003 | Damaging | possibly damaging | 0.895 | possibly damaging | 0.615 | effect | 17 | 4.25 | HIGH | 91 | probably begign | -3.77 | Deleterious | 0.792719 | damaging | 0.790752 | damaging | 0.701298 | damaging | Green et al (1989) |
| 18 | Mild | NS | c.501G>T | p.Gln167His | 0.03 | Damaging | benign | 0.064 | benign | 0.013 | neutral | -53 | 4.25 | HIGH | 176 | probably begign | -1.06 | Neutral | 0.756559 | damaging | 0.751652 | damaging | 0.645482 | damaging | Van de Water et al (1996) |
| <1 | Severe | S | c.505T>C | p.Ser169Pro | 0.005 | Damaging | probably damaging | 1 | probably damaging | 0.981 | effect | 58 | 3.705 | HIGH | 361 | possibly damaging | -3.61 | Deleterious | 0.761003 | damaging | 0.757789 | damaging | 0.654243 | damaging | Saad et al (1994) |
| 15 | Mild | NS | c.506C>G | p.Ser169Cys | 0.002 | Damaging | probably damaging | 1 | probably damaging | 0.993 | effect | 30 | 4.245 | HIGH | 361 | possibly damaging | -3.77 | Deleterious | 0.824296 | damaging | 0.821794 | damaging | 0.74561 | damaging | Vidal et al (2000) |
| <1 | Severe | S | c.507C>T | p.Cys170Phe | 1 | Tolerated | probably damaging | 1 | probably damaging | 1 | effect | 69 | 4.245 | HIGH | 361 | possibly damaging | 0 | Neutral | 0.590335 | damaging | 0.598413 | damaging | 0.650794 | damaging | Wulff et al (1998) |
| <1 | Severe | S | c.508T>C | p.Cys170Arg | 0.001 | Damaging | probably damaging | 1 | probably damaging | 1 | effect | 78 | 4.245 | HIGH | 750 | probably damaging | -10.14 | Deleterious | 0.429916 | benign | 0.427287 | bening | 0.406511 | bening | Belvini et al (2005) |
| <1 | Severe | S | c.509G>A | p.Cys170Tyr | 0.001 | Damaging | probably damaging | 1 | probably damaging | 1 | effect | 69 | 3.865 | HIGH | 750 | probably damaging | -9.32 | Deleterious | 0.460605 | benign | 0.470067 | bening | 0.446962 | bening | Belvini et al (2005) |
| <1 | Severe | S | c.509G>C | p.Cys170Ser | 0.002 | Damaging | probably damaging | 1 | probably damaging | 1 | effect | 71 | 3.315 | HIGH | 750 | probably damaging | -8.46 | Deleterious | 0.295058 | benign | 0.31186 | bening | 0.445181 | bening | Li et al (2000) |
| <1 | Severe | S | c.277G>A | p.Asp93Asn | 0 | Damaging | possibly damaging | 0.952 | possibly damaging | 0.852 | effect | 9 | 2.07 | MEDIUM | 750 | probably damaging | -4.22 | Deleterious | 0.908384 | damaging | 0.907092 | damaging | 0.867373 | damaging | Miller et al (2012) |
| <1 | Severe | S | c.277G>C | p.Asp93His | 0 | Damaging | probably damaging | 0.986 | probably damaging | 0.957 | effect | 21 | 2.615 | MEDIUM | 750 | probably damaging | -5.9 | Deleterious | 0.867194 | damaging | 0.864692 | damaging | 0.806848 | damaging | Mahajan et al (2007) |
| 10 | Mild | NS | c.278A>G | p.Asp93Gly | 0 | Damaging | possibly damaging | 0.666 | possibly damaging | 0.573 | neutral | -9 | 1.765 | LOW | 750 | probably damaging | -5.9 | Deleterious | 0.918889 | damaging | 0.917033 | damaging | 0.881565 | damaging | Davis et al (1987) |
| 14 | Mild | NS | c.279T>A | p.Asp93Glu | 0 | Damaging | possibly damaging | 0.833 | possibly damaging | 0.583 | effect | 6 | 2.16 | MEDIUM | 750 | probably damaging | -3.37 | Deleterious | 0.940023 | damaging | 0.938921 | damaging | 0.912809 | damaging | Bottema et al (1990) |
| 25 | Mild | NS | c.280G>A | p.Gly94Arg | 0.001 | Damaging | probably damaging | 0.999 | probably damaging | 0.93 | effect | 80 | 1.1 | LOW | 750 | probably damaging | -6.26 | Deleterious | 0.907139 | damaging | 0.904999 | damaging | 0.864386 | damaging | Attali et al (1999) |
| 18 | Mild | NS | c.281G>T | p.Gly94Val | 0.001 | Damaging | probably damaging | 0.995 | possibly damaging | 0.852 | effect | 62 | -0.615 | NEUTRAL | 750 | probably damaging | -7.1 | Deleterious | 0.830139 | damaging | 0.826139 | damaging | 0.751814 | damaging | Costa et al (2000) |
| 4 | Moderate | S | c.284A>G | p.Asp95Gly | 0.006 | Damaging | probably damaging | 0.978 | possibly damaging | 0.817 | effect | 36 | 2.71 | MEDIUM | 176 | probably begign | -4.42 | Deleterious | 0.892089 | damaging | 0.889586 | damaging | 0.842384 | damaging | Thorland et al (1995) |
| >5 | Mild | NS | c.283G>T | p.Asp95Tyr | 0.001 | Damaging | probably damaging | 1 | probably damaging | 0.952 | effect | 36 | 3.3 | MEDIUM | 176 | probably begign | -6.13 | Deleterious | 0.831096 | damaging | 0.82714 | damaging | 0.753242 | damaging | Costa et al (2000) |
| <1 | Severe | S | c.286C>G | p.Gln96Glu | 0 | Damaging | possibly damaging | 0.871 | benign | 0.337 | effect | 45 | -1.7 | NEUTRAL | 750 | probably damaging | -2.56 | Deleterious | 0.889429 | damaging | 0.886882 | damaging | 0.838524 | damaging | Quadros et al, (2009) |
| <1 | Severe | S | c.287A>C | p.Gln96Pro | 0 | Damaging | benign | 0.066 | benign | 0.087 | effect | 49 | 0.15 | NEUTRAL | 750 | probably damaging | -5.11 | Deleterious | 0.861696 | damaging | 0.858479 | damaging | 0.797978 | damaging | Lozier et al (1990) |
| 24 | Mild | NS | c.288G>C | p.Gln96His | 0 | Damaging | possibly damaging | 0.691 | benign | 0.415 | effect | 5 | 0.98 | LOW | 750 | probably damaging | -4.26 | Deleterious | 0.845641 | damaging | 0.842349 | damaging | 0.774953 | damaging | Saad et al (1994) |
| 15 | Mild | NS | c.288G>T | p.Gln96His | 0 | Damaging | possibly damaging | 0.691 | benign | 0.415 | effect | 5 | 0.98 | LOW | 750 | probably damaging | -4.26 | Deleterious | 0.886318 | damaging | 0.883775 | damaging | 0.834089 | damaging | Centre B21 (unpublished) |
| <1 | Severe | S | c.289T>C | p.Cys97Arg | 0 | Damaging | probably damaging | 1 | probably damaging | 0.999 | effect | 86 | 4.755 | HIGH | 750 | probably damaging | -10.23 | Deleterious | 0.685597 | damaging | 0.693285 | damaging | 0.786223 | damaging | Costa et al (2000) |
| <1 | Severe | S | c.290G>A | p.Cys97Tyr | 0 | Damaging | probably damaging | 1 | probably damaging | 0.999 | effect | 78 | 4.41 | HIGH | 750 | probably damaging | -9.37 | Deleterious | 0.694314 | damaging | 0.701566 | damaging | 0.798045 | damaging | Centre B14 (unpublished) |
| <1 | Severe | S | c.291T>G | p.Cys97Trp | 0 | Damaging | probably damaging | 1 | probably damaging | 1 | effect | 73 | 4.755 | HIGH | 750 | probably damaging | -9.37 | Deleterious | 0.665021 | damaging | 0.672262 | damaging | 0.756213 | damaging | Attali et al (1999) |
| >5 | Mild | NS | c.301C>A | p.Pro101Thr | 0 | Damaging | probably damaging | 1 | probably damaging | 0.999 | effect | 43 | 3.535 | HIGH | 750 | probably damaging | -6.55 | Deleterious | 0.491514 | benign | 0.511901 | bening | 0.50668 | damaging | Miller et al (2012) |
| >5 | Mild | NS | c.301C>G | p.Pro101Ala | 0 | Damaging | probably damaging | 1 | probably damaging | 0.996 | effect | 30 | 3.38 | MEDIUM | 750 | probably damaging | -6.55 | Deleterious | 0.941465 | damaging | 0.940403 | damaging | 0.914926 | damaging | Miller et al (2012) |
| 12 | Mild | NS | c.301C>T | p.Pro101Ser | 0 | Damaging | probably damaging | 1 | probably damaging | 0.999 | effect | 43 | 3.58 | HIGH | 750 | probably damaging | -6.52 | Deleterious | 0.887416 | damaging | 0.88508 | damaging | 0.835951 | damaging | Green et al (1991) |
| 6 | Mild | NS | c.302C>A | p.Pro101Gln | 0 | Damaging | probably damaging | 1 | probably damaging | 0.997 | effect | 20 | 4.185 | HIGH | 750 | probably damaging | -6.35 | Deleterious | 0.912532 | damaging | 0.910803 | damaging | 0.872671 | damaging | Ketterling et al (1993) |
| 22 | Mild | NS | c.302C>G | p.Pro101Arg | 0 | Damaging | probably damaging | 1 | probably damaging | 0.999 | effect | 35 | 3.535 | HIGH | 750 | probably damaging | -7.3 | Deleterious | 0.849056 | damaging | 0.847042 | damaging | 0.781652 | damaging | Montejo et al (1999) |
| 26 | Mild | NS | c.302C>T | p.Pro101Leu | 0 | Damaging | probably damaging | 1 | probably damaging | 1 | effect | 19 | 3.49 | MEDIUM | 750 | probably damaging | -8.22 | Deleterious | 0.829743 | damaging | 0.827508 | damaging | 0.753767 | damaging | Green et al (1991) |
| 1 | Moderate | S | c.304T>A | p.Cys102Ser | 0 | Damaging | probably damaging | 1 | probably damaging | 1 | effect | 55 | 4.465 | HIGH | 911 | probably damaging | -8.52 | Deleterious | 0.14652 | benign | 0.15637 | bening | 0.223219 | bening | Tartary et al (1993) |
| <1 | Severe | S | c.304T>C | p.Cys102Arg | 0 | Damaging | probably damaging | 1 | probably damaging | 1 | effect | 68 | 4.26 | HIGH | 911 | probably damaging | -10.23 | Deleterious | 0.874032 | damaging | 0.871539 | damaging | 0.816622 | damaging | Miller et al (2012) |
| <5 | Moderate | S | c.305G>A | p.Cys102Tyr | 0 | Damaging | probably damaging | 1 | probably damaging | 1 | effect | 65 | 4.12 | HIGH | 911 | probably damaging | -9.37 | Deleterious | 0.867071 | damaging | 0.864695 | damaging | 0.806852 | damaging | Yu et al (2012) |
| <1 | Severe | S | c.305G>C | p.Cys102Ser | 0 | Damaging | probably damaging | 1 | probably damaging | 1 | effect | 55 | 4.465 | HIGH | 911 | probably damaging | -8.52 | Deleterious | 0.888615 | damaging | 0.886475 | damaging | 0.837943 | damaging | Ludwig et al (1991) |
| <1 | Severe | S | c.305G>T | p.Cys102Phe | 0 | Damaging | probably damaging | 1 | probably damaging | 1 | effect | 63 | 4.81 | HIGH | 911 | probably damaging | -9.37 | Deleterious | 0.888615 | damaging | 0.886475 | damaging | 0.837943 | damaging | Radic et al (2013) |
| 10 | Mild | NS | c.312T>G | p.Asn104Lys | 0.012 | Damaging | probably damaging | 1 | probably damaging | 0.969 | effect | 43 | 3.975 | HIGH | 750 | probably damaging | -4.51 | Deleterious | 0.33185 | benign | 0.337523 | bening | 0.257754 | bening | Centre B31 (unpublished) |
| 20 | Mild | NS | c.313G>A | p.Gly105Ser | 0.071 | Tolerated | possibly damaging | 0.943 | possibly damaging | 0.837 | neutral | -34 | 3.02 | MEDIUM | 750 | probably damaging | -2.61 | Deleterious | 0.814312 | damaging | 0.811411 | damaging | 0.730789 | damaging | Costa et al (2000) |
| 2 | Moderate | S | c.314G>T | p.Gly105Val | 0.015 | Damaging | probably damaging | 0.995 | probably damaging | 0.978 | effect | 44 | 4.54 | HIGH | 750 | probably damaging | -4.98 | Deleterious | 0.771257 | damaging | 0.766717 | damaging | 0.666988 | damaging | Bottema et al (1991) |
| >5 | Mild | S | c.314G>A | p.Gly105Asp | 0.241 | Tolerated | benign | 0.143 | benign | 0.213 | neutral | -22 | 2.385 | MEDIUM | 750 | probably damaging | -3.05 | Deleterious | 0.831381 | damaging | 0.82893 | damaging | 0.755797 | damaging | Costa et al (2000) |
| 11 | Mild | NS | c.316G>A | p.Gly106Ser | 0.004 | Damaging | probably damaging | 1 | probably damaging | 0.999 | effect | 26 | 2.49 | MEDIUM | 750 | probably damaging | -4.65 | Deleterious | 0.806147 | damaging | 0.802996 | damaging | 0.718776 | damaging | Radic et al (2013) |
| <1 | Severe | S | c.316G>C | p.Gly106Arg | 0.001 | Damaging | probably damaging | 1 | probably damaging | 1 | effect | 42 | 3.935 | HIGH | 750 | probably damaging | -6.35 | Deleterious | 0.821177 | damaging | 0.819281 | damaging | 0.742023 | damaging | Miller et al (2012) |
| <1 | Severe | S | c.316G>T | p.Gly106Cys | 0.001 | Damaging | probably damaging | 1 | probably damaging | 1 | effect | 43 | 4.48 | HIGH | 750 | probably damaging | -7.07 | Deleterious | 0.150759 | benign | 0.163145 | bening | 0.23289 | bening | Elmahmoudi et al (2011) |
| <1 | Severe | S | c.317G>A | p.Gly106Asp | 0.001 | Damaging | probably damaging | 1 | probably damaging | 1 | effect | 70 | 4.135 | HIGH | 750 | probably damaging | -5.63 | Deleterious | 0.94557 | damaging | 0.94479 | damaging | 0.921188 | damaging | Miller et al (2012) |
| <1 | Severe | S | c.316G>C | p.Gly106Arg | 0.001 | Damaging | probably damaging | 1 | probably damaging | 1 | effect | 42 | 3.935 | HIGH | 750 | probably damaging | -6.35 | Deleterious | 0.92802 | damaging | 0.9271 | damaging | 0.895935 | damaging | Bottema et al (1991) |
| 1 | Moderate | S | c.323G>A | p.Cys108Tyr | 0 | Damaging | probably damaging | 1 | probably damaging | 1 | effect | 87 | 4.84 | HIGH | 750 | probably damaging | -9.37 | Deleterious | 0.94852 | damaging | 0.947812 | damaging | 0.925501 | damaging | Thorland et al (1995) |
| <1 | Severe | S | c.323G>C | p.Cys108Ser | 0 | Damaging | probably damaging | 1 | probably damaging | 1 | effect | 81 | 4.495 | HIGH | 750 | probably damaging | -8.52 | Deleterious | 0.92802 | damaging | 0.9271 | damaging | 0.895935 | damaging | Montejo et al (1999) |
| <1 | Severe | S | c.323G>T | p.Cys108Phe | 0 | Damaging | probably damaging | 1 | probably damaging | 1 | effect | 85 | 4.84 | HIGH | 750 | probably damaging | -9.37 | Deleterious | 0.94852 | damaging | 0.947812 | damaging | 0.925501 | damaging | Belvini et al (2005) |
| <1 | Severe | S | c.324C>G | p.Cys108Trp | 0 | Damaging | probably damaging | 1 | probably damaging | 1 | effect | 88 | 4.84 | HIGH | 750 | probably damaging | -9.37 | Deleterious | 0.096067 | benign | 0.105233 | bening | 0.150221 | bening | Belvini et al (2005) |
| 3 | Moderate | S | c.328G>A | p.Asp110Asn | 0.006 | Damaging | probably damaging | 0.997 | probably damaging | 0.957 | effect | 60 | 1.92 | MEDIUM | 362 | possibly damaging | -4.21 | Deleterious | 0.448723 | benign | 0.478876 | bening | 0.683596 | damaging | Winship and Dragon (1991) |
| 8 | Mild | NS | c.329A>G | p.Asp110Gly | 0.034 | Damaging | possibly damaging | 0.929 | possibly damaging | 0.905 | effect | 20 | 2.645 | MEDIUM | 362 | possibly damaging | -5.91 | Deleterious | 0.469487 | benign | 0.499381 | bening | 0.712868 | damaging | Green et al (1989) |
| 12 | Mild | NS | c.330T>G | p.Asp110Glu | 0.002 | Damaging | probably damaging | 0.962 | possibly damaging | 0.768 | effect | 40 | 2.05 | MEDIUM | 362 | possibly damaging | -3.29 | Deleterious | 0.378273 | benign | 0.403293 | bening | 0.575702 | damaging | Tartary et al (1993) |
| >5 | Mild | NS | c.329A>T | p.Asp110Val | 0.001 | Damaging | probably damaging | 0.999 | probably damaging | 0.996 | effect | 53 | 2.925 | MEDIUM | 362 | possibly damaging | -7.58 | Deleterious | 0.92052 | damaging | 0.919135 | damaging | 0.884565 | damaging | Rydz et al (2013) |
| 26 | Mild | NS | c.335T>A | p.Ile112Asn | 0.001 | Damaging | probably damaging | 0.972 | probably damaging | 0.924 | effect | 65 | 1.27 | LOW | 176 | probably begign | -4.49 | Deleterious | 0.91277 | damaging | 0.911198 | damaging | 0.873235 | damaging | Enayat et al (2004) |
| 20 | Mild | NS | c.335T>C | p.Ile112Thr | 0.032 | Damaging | benign | 0.335 | benign | 0.371 | effect | 15 | 1.505 | LOW | 176 | probably begign | -2.82 | Deleterious | 0.414133 | benign | 0.442737 | bening | 0.632007 | damaging | Tartary et al (1993) |
| <1 | Severe | S | c.339T>A | p.Asn113Lys | 0.016 | Damaging | benign | 0.147 | benign | 0.042 | neutral | -28 | 2.44 | MEDIUM | 324 | possibly damaging | -1.98 | Neutral | 0.393644 | benign | 0.400701 | bening | 0.347942 | bening | Ketterling et al (1999) |
| <5 | Moderate | S | c.340T>C | p.Ser114Pro | 0.026 | Damaging | benign | 0.026 | benign | 0.032 | neutral | 0 | 3.455 | MEDIUM | 176 | probably begign | -2.49 | Neutral | 0.93977 | damaging | 0.93885 | damaging | 0.912709 | damaging | Yu et al (2012) |
| <1 | Severe | S | c.343T>A | p.Tyr115Asn | 0 | Damaging | probably damaging | 1 | probably damaging | 0.999 | effect | 79 | 3.78 | HIGH | 750 | probably damaging | -7.67 | Deleterious | 0.942654 | damaging | 0.941803 | damaging | 0.916924 | damaging | Ketterling et al (1999) |
| <1 | Severe | S | c.344A>G | p.Tyr115Cys | 0 | Damaging | probably damaging | 1 | probably damaging | 1 | effect | 58 | 4.035 | HIGH | 750 | probably damaging | -7.67 | Deleterious | 0.94557 | damaging | 0.94479 | damaging | 0.921188 | damaging | Radic et al (2013) |
| <1 | Severe | S | c.349T>A | p.Cys117Ser | 0 | Damaging | probably damaging | 1 | probably damaging | 1 | effect | 73 | 4.845 | HIGH | 797 | probably damaging | -8.52 | Deleterious | 0.865359 | damaging | 0.863014 | damaging | 0.804453 | damaging | Nielsen et al (1995) |
| <1 | Severe | S | c.349T>C | p.Cys117Arg | 0 | Damaging | probably damaging | 1 | probably damaging | 1 | effect | 84 | 4.5 | HIGH | 797 | probably damaging | -10.23 | Deleterious | 0.920232 | damaging | 0.91883 | damaging | 0.884129 | damaging | Jayandharan et al (2005) |
| <1 | Severe | S | c.350G>A | p.Cys117Tyr | 0 | Damaging | probably damaging | 1 | probably damaging | 1 | neutral | -68 | 4.845 | HIGH | 797 | probably damaging | -9.37 | Deleterious | 0.900232 | damaging | 0.898347 | damaging | 0.85489 | damaging | Belvini et al (2005) |
| <1 | Severe | S | c.350G>T | p.Cys117Phe | 0 | Damaging | probably damaging | 1 | probably damaging | 1 | neutral | -2 | 4.845 | HIGH | 797 | probably damaging | -9.37 | Deleterious | 0.907315 | damaging | 0.905587 | damaging | 0.865226 | damaging | Jayandharan et al (2005) |
| <1 | Severe | S | c.351T>G | p.Cys117Trp | 0 | Damaging | probably damaging | 1 | probably damaging | 1 | effect | 61 | 4.845 | HIGH | 797 | probably damaging | -9.37 | Deleterious | 0.900254 | damaging | 0.898678 | damaging | 0.855362 | damaging | Centre B14 (unpublished) |
| 1 | Moderate | S | c.355T>C | p.Cys119Arg | 0 | Damaging | probably damaging | 1 | probably damaging | 1 | effect | 10 | 4.815 | HIGH | 750 | probably damaging | -10.23 | Deleterious | 0.886707 | damaging | 0.885296 | damaging | 0.836259 | damaging | David et al (1998) |
| <1 | Severe | S | c.356G>A | p.Cys119Tyr | 0 | Damaging | probably damaging | 1 | probably damaging | 1 | neutral | -60 | 4.815 | HIGH | 750 | probably damaging | -9.37 | Deleterious | 0.85282 | damaging | 0.850057 | damaging | 0.785956 | damaging | Weinmann et al (1998) |
| <1 | Severe | S | c.356G>T | p.Cys119Phe | 0 | Damaging | probably damaging | 1 | probably damaging | 1 | neutral | -44 | 4.815 | HIGH | 750 | probably damaging | -9.37 | Deleterious | 0.863958 | damaging | 0.861224 | damaging | 0.801896 | damaging | Wulff et al (1995) |
| 14 | Mild | NS | c.364G>A | p.Gly122Arg | 0.07 | Tolerated | probably damaging | 1 | probably damaging | 0.997 | effect | 64 | 3.26 | MEDIUM | 750 | probably damaging | -4.92 | Deleterious | 0.88189 | damaging | 0.879712 | damaging | 0.828289 | damaging | Centre B31 (unpublished) |
| 6 | Mild | NS | c.365G>T | p.Gly122Val | 0.001 | Damaging | probably damaging | 1 | probably damaging | 1 | effect | 60 | 4.68 | HIGH | 750 | probably damaging | -6.33 | Deleterious | 0.772578 | damaging | 0.770541 | damaging | 0.672447 | damaging | Saad et al (1994) |
| 10 | Mild | NS | c.368T>C | p.Phe123Ser | 0.001 | Damaging | probably damaging | 1 | probably damaging | 0.981 | effect | 64 | 3.11 | MEDIUM | 176 | probably begign | -5.63 | Deleterious | 0.693254 | damaging | 0.714104 | damaging | 0.795325 | damaging | Weinmann et al (1998) |
| <1 | Severe | S | c.368T>G | p.Phe123Cys | 0 | Damaging | probably damaging | 1 | probably damaging | 0.997 | effect | 58 | 3.8 | HIGH | 176 | probably begign | -5.63 | Deleterious | 0.636654 | damaging | 0.64114 | damaging | 0.711786 | damaging | Enayat et al (2004) |
| 19 | Mild | NS | c.369T>G | p.Phe123Leu | 0.005 | Damaging | possibly damaging | 0.954 | possibly damaging | 0.67 | effect | 66 | 2.65 | MEDIUM | 176 | probably begign | -4.32 | Deleterious | 0.368987 | benign | 0.387187 | bening | 0.55271 | damaging | Ljung et al (2001) |
| <1 | Severe | S | c.370G>A | p.Glu124Lys | 0.051 | Tolerated | probably damaging | 0.993 | possibly damaging | 0.78 | neutral | -3 | 1.01 | LOW | 324 | possibly damaging | -2.23 | Neutral | 0.857525 | damaging | 0.855001 | damaging | 0.793014 | damaging | Onay et al (2003) |
| <1 | Severe | S | c.373G>A | p.Gly125Arg | 0.002 | Damaging | probably damaging | 1 | probably damaging | 1 | effect | 85 | 4.455 | HIGH | 750 | probably damaging | -6.71 | Deleterious | 0.831048 | damaging | 0.827478 | damaging | 0.753725 | damaging | Costa et al (2000) |
| <1 | Severe | S | c.383G>A | p.Cys128Tyr | 0 | Damaging | probably damaging | 1 | probably damaging | 1 | effect | 63 | 4.45 | HIGH | 750 | probably damaging | -9.38 | Deleterious | 0.562398 | damaging | 0.566664 | damaging | 0.584854 | damaging | Li et al (2000) |
| <1 | Severe | S | c.383G>C | p.Cys128Ser | 0 | Damaging | probably damaging | 1 | probably damaging | 1 | effect | 74 | 4.45 | HIGH | 750 | probably damaging | -8.53 | Deleterious | 0.917615 | damaging | 0.91615 | damaging | 0.880304 | damaging | Belvini et al (2005) |
| <1 | Severe | S | c.385G>A | p.Glu129Lys | 0.002 | Damaging | probably damaging | 1 | probably damaging | 0.961 | effect | 64 | 3.02 | MEDIUM | 750 | probably damaging | -3.18 | Deleterious | 0.885854 | damaging | 0.88393 | damaging | 0.83431 | damaging | Centre B15 (unpublished) |
| >5 | Mild | NS | c.386A>G | p.Glu129Gly | 0.001 | Damaging | probably damaging | 1 | probably damaging | 0.98 | effect | 58 | 2.815 | MEDIUM | 750 | probably damaging | -5.77 | Deleterious | 0.874457 | damaging | 0.87275 | damaging | 0.81835 | damaging | Konkle et al (2014) |

***, Categories based on clinical phenotype as: Severe (FIX:C 0<1%), Moderate (FIX:C 1≤5%), Mild (FIX:C >5%).**

****, Categories assumed in this work (see the text): Severe/Moderate (S, FIX:C 0≤5%), Non-severe (NS, FIX:C>5%).**

***gP*: group prediction, *wgP6*: weighting group prediction with 6 programs, *wgP4*: weighting group prediction with 4 programs.**
